# Hepatocyte CBX2 Safeguards Hepatic Lipid Homeostasis by Elevating Ca^2+^‐CaMKK2‐AMPK Signaling to Counter MASLD

**DOI:** 10.1002/advs.78372

**Published:** 2026-10-18

**Authors:** Xiang Wei, Li Lin, Xiuyun Wang, Hui Du, Menglong Zhang, Kechao Tang, Han Gao, Xinrui Xie, Jingya Gao, Xu Wang, Zhenglin Wang, Siwei Huang, Lei Zhang, Fang Gu, Gong‐Hong Wei, Yinliang Qi, Wei Chen, Yongsheng Chang, Huabing Zhang

**Affiliations:** ^1^ Department of Biochemistry and Molecular Biology Metabolic Disease Research Center School of Basic Medicine Anhui Medical University Hefei China; ^2^ Department of Hyperbaric Oxygen The Second People's Hospital of Hefei Hefei Hospital Affiliated to Anhui Medical University Hefei China; ^3^ MOE Key Laboratory of Metabolism and Molecular Medicine & Department of Biochemistry and Molecular Biology, School of Basic Medical Sciences, and Fudan University Shanghai Cancer Center Shanghai Medical College of Fudan University Shanghai China; ^4^ Department of Anesthesiology Division of Life Sciences and Medicine The First Affiliated Hospital of USTC University of Science and Technology of China Hefei China; ^5^ Department of General Surgery The First Affiliated Hospital of Anhui Medical University Hefei Anhui China; ^6^ Department of Endocrinology and Metabolism Nanfang Hospital Southern Medical University Guangzhou China; ^7^ State Key Laboratory of Common Mechanism Research for Major Diseases Suzhou Institute of Systems Medicine Chinese Academy of Medical Sciences & Peking Union Medical College Suzhou China; ^8^ Tianjin Key Laboratory of Cellular Homeostasis and Disease, Key Department of Physiology and Pathophysiology, Laboratory of Immune Microenvironment and Disease (Ministry of Education) Tianjin Medical University Tianjin China; ^9^ Department of Otorhinolaryngology Head and Neck Surgery The First Affiliated Hospital of Anhui Medical University Hefei China

**Keywords:** AMPK, CaMKK2, CBX2, fatty‐acid oxidation, inflammation, lipogenesis, MASLD/MASH

## Abstract

Metabolic dysfunction‐associated steatotic liver disease (MASLD) affects approximately one‐third of adults and lacks effective pharmacological therapies. Here, we identify Chromobox 2 (CBX2), a Polycomb‐associated chromatin regulator, as a previously unrecognized regulator of hepatic lipid homeostasis. CBX2 expression is markedly reduced in hepatocytes from patients with MASLD, multiple mouse models (*db/db*, *ob/ob*, HFD, and HFHC), and steatotic hepatocytes *in* *vitro*. Hepatocyte‐specific CBX2 overexpression ameliorates hepatic steatosis, improves glucose metabolism, and suppresses inflammation, whereas CBX2 deficiency exacerbates these metabolic disturbances. Mechanistically, CBX2 enhances H3K4me3 enrichment at the *Orai1* promoter, promoting *Orai1* transcription and increasing intracellular Ca^2+^ levels. Elevated Ca^2+^ activates the CaMKK2‐AMPK signaling pathway, leading to ACC phosphorylation, inhibition of *de novo* lipogenesis (FAS, ACC, and SCD‐1), enhancement of fatty acid oxidation (PPARα and CPT‐1a), and suppression of NF‐κB/JNK‐mediated inflammation. Genetic and pharmacological interventions further establish the necessity of this Ca^2+^‐CaMKK2‐AMPK axis for CBX2‐mediated metabolic protection. Collectively, our findings reveal a CBX2‐driven metabolic regulatory circuit and highlight CBX2 and its downstream signaling pathway as potential therapeutic targets for MASLD.

## Graphical abstract

In hepatocytes, CBX2 promotes H3K4me3 enrichment at the *Orai1* promoter to enhance *Orai1* transcriptional activation, thereby increasing intracellular Ca^2^^+^ levels. The elevated Ca^2^^+^ activates the CaMKK2‐AMPK signaling cascade, resulting in ACC phosphorylation and downstream metabolic regulation. This cascade suppresses *de novo* lipogenesis (*Fasn*, *Acc*, *Scd‐1*), enhances fatty‐acid oxidation (PPARα, CPT‐1a), and attenuates inflammatory signaling (reduced NF‐κB and JNK phosphorylation), thereby lowering triglyceride accumulation and macrophage infiltration and improving insulin signaling (AKT/GSK‐3β). Pharmacologic and genetic perturbations (Ca^2^^+^ chelation or supplementation; CaMKK2 inhibitor STO‐609; AMPK inhibitor Compound C; AMPK activator A‐769662) validate the pathway's necessity and sufficiency in primary hepatocytes and in vivo. Overall, CBX2 counters steatosis, insulin resistance, and inflammation in HFD and HFHC models and rescues metabolic defects in *Cbx2^LKO^* livers through this Ca^2^^+^‐CaMKK2‐AMPK mechanism.

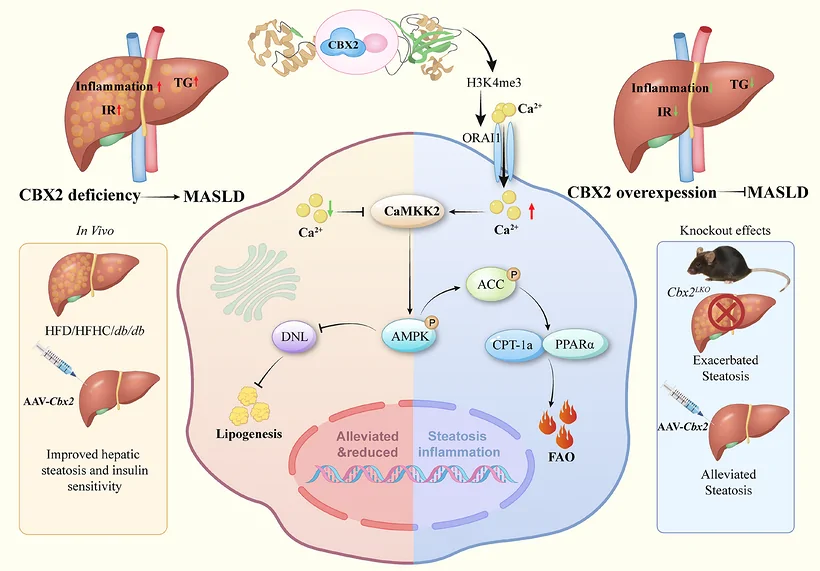

## 1 Introduction

Metabolic dysfunction‐associated steatotic liver disease (MASLD) represents a hepatic manifestation of systemic metabolic imbalance and has emerged as the most prevalent form of chronic liver disease globally [1]. Characterized by hepatic lipid content exceeding 5% of liver mass, without appropriate intervention, MASLD follows a progressive trajectory, advancing from simple steatosis to metabolic dysfunction‐associated steatohepatitis (MASH) and even fibrosis [2]. Current estimates indicate that MASLD affects approximately one‐third of the global population, with its burden continuing to expand alongside the worldwide escalation of obesity and type 2 diabetes mellitus (T2DM) [3, 4]. In addition to hepatic complications, MASLD is associated with a markedly increased risk of cardiovascular disease, chronic kidney disease, and multiple forms of extrahepatic malignancy [4]. Despite emerging pharmacothrapies such as resmetirom, recently approved for stage 2–3 fibrosis‐sustained lifestyle modification remains the mainstay of treatment [5, 6]. However, given the limited efficacy and accessibility of current interventions, there is a pressing requirement to elucidate the molecular determinants driving MASLD onset and progression to inform new therapeutic strategies.

Excessive lipid accumulation in hepatocytes is a central event in MASLD pathogenesis [7]. Dietary and environmental factors, together with obesity‐induced insulin resistance (IR), increased circulating levels of free fatty acids (FFA) and cholesterol, contribute substantially to disease pathogenesis [8]. Their increased hepatic uptake, together with enhanced *de novo* lipogenesis (DNL), leads to triglyceride (TG) overproduction and deposition [9]. The accumulation of lipotoxic intermediates disrupts mitochondrial function and enhances oxidative stress, leading to excessive production of reactive oxygen species that impair cellular organelles and activate endoplasmic reticulum stress pathways [9, 10]. These processes activate inflammatory and cell‐death pathways that perpetuate hepatocellular injury. The maintenance of lipid homeostasis therefore relies on an intricate regulatory network that integrates nutrient signals, transcriptional control, and energy‐sensing kinases such as AMP‐activated protein kinase (AMPK). Yet, the upstream regulators that couple chromatin state to metabolic signaling in MASLD remain largely unexplored.

Epigenetic regulation is increasingly recognized as a key mechanism linking metabolic stress to gene expression reprogramming. Polycomb group proteins, assembled into Polycomb repressive complex 1 and 2 (PRC1/2), are canonical chromatin modifiers that enforce transcriptional silencing through histone methylation and chromatin compaction [11, 12]. PRC1, in particular, catalyzes H2A lysine 119 ubiquitination and cooperates with PRC2‐mediated H3K27 trimethylation to establish repressive chromatin domains [13]. Chromobox homolog 2 (CBX2), a core component of canonical PRC1, binds to H3K27me3 via its chromodomain and recruits the PRC1 complex to target loci, thereby stabilizing silenced chromatin states [14]. In addition to its repressive function, CBX2 has been shown to act as a context‐dependent transcriptional activator [15]. Previous work has mainly implicated CBX2 in tumorigenesis, where it promotes cell proliferation and cell‐cycle progression [16, 17]. Other CBX family members exhibit metabolic functions‐CBX4 enhances adipose thermogenesis through PRDM16 sumoylation [18], while CBX7 restricts adipocyte differentiation [19]‐suggesting a broader role of the Polycomb system in energy metabolism. However, whether and how CBX2 contributes to hepatic metabolic homeostasis remains unknown.

Here, we identify CBX2 as a previously unrecognized regulator of hepatic lipid metabolism. We show that CBX2 expression is markedly reduced in hepatocytes from patients with MASLD and in multiple experimental mouse models. Gain‐ and loss‐of‐function studies demonstrate that CBX2 restrains hepatic steatosis and inflammation, as its overexpression ameliorates, whereas hepatocyte‐specific deletion aggravates, lipid accumulation in high‐fat diet (HFD) and high‐fat/high‐cholesterol (HFHC) models. Mechanistically, CBX2 governs hepatic lipid metabolism and inflammatory responses through modulation of the AMPK signaling axis, thereby linking Polycomb‐mediated chromatin regulation to metabolic signaling. These findings reveal a mechanistic connection between CBX2‐mediated regulation and hepatic energy metabolism and highlight CBX2 as a potential therapeutic target in MASLD.

## 2 Experimental Section

### 2.1 Clinicopathological Specimen Preparation

Pathological specimens from patients with MASLD and healthy controls were obtained from the Department of Pathology, the First Affiliated Hospital of Anhui Medical University. These samples used in the present study were obtained from patients who underwent clinical examination at the First Affiliated Hospital of Anhui Medical University. Patients from whom normal liver histological sections were obtained were diagnosed without liver diseases, and histopathological examination confirmed the absence of pathological changes in the liver tissues. Therefore, these samples were considered normal liver tissue and were used as controls in this study. Meanwhile, liver tissue sections from patients diagnosed with MASLD were used as the experimental group in the study. Histological assessments were performed independently by two experienced pathologists who were blinded to all clinical data. All human‐derived specimens were obtained and handled in compliance with the principles of the Declaration of Helsinki. All tissue samples were collected following informed consent from the patients and the study protocol was approved by the Research Ethics Committee of Anhui Medical University (Approval No. 83230145).

### 2.2 Animals and Treatments

Male *db/db*, *ob/ob*, *db/m* and *C57BL/6J* aged 4 weeks were purchased from GemPharmatech (Nanjing, China). After overnight fasting, *C57BL/6J* were fed either a high‐fat diet (SYSE BIO, 60 kcal% fat, PD6001) or a high‐fat and high‐cholesterol diet (SYSE BIO, 17% protein, 43% carbohydrate, 41% fat, and 0.21% cholesterol, D12079B) to induce MASLD. After 2 months of dietary intervention, Mice were randomly allocated to undergo a single administration of recombinant adeno‐associated virus (AAV‐*Gfp* or AAV‐*Cbx2*, 2 × 10^1^ ^1^ vector genomes (vg) per mouse). Animals were maintained on their respective diets for an additional 2 months before subsequent analyses. *db/db* mice were fed a normal diet, all other procedures were as described previously. All animal procedures were reviewed and approved by the Institutional Animal Care and Use Committee of Anhui Medical University and were performed in compliance with the ARRIVE guidelines (Approval No. LLSC20230493).

### 2.3 Construction of *Cbx2^LKO^* Mice

Hepatocyte‐restricted deletion of Cbx2 (*Cbx2^LKO^*) was achieved by crossing mice harboring loxP‐flanked Cbx2 alleles (*Cbx2* ^flox/flox^) with *C57BL/6JGpt*‐Rosa26^em1Cin (Alb‐iCre‐polyA)^/Gpt mice. The *Cbx2* ^flox/flox^ (Strain ID: T008509) and *C57BL/6JGpt*‐Rosa26^em1Cin (Alb‐iCre‐polyA)^/Gpt mice (Strain ID: T054834) were obtained from GemPharmatech (Nanjing, China). The Cbx2 gene has two transcripts. Based on the gene structure, exons 3–4 of the *Cbx2*‐201 transcript (ENSMUST00000026662.7), which include a 172 bp coding sequence, were selected as the knockout regions. Using the CRISPR/Cas9 genome‐editing system, donor vectors were microinjected into fertilized *C57BL/6JGpt* mouse zygotes. Following manipulation, embryos were surgically transferred into hormonally prepared recipient females, from which F0 founder mice were generated and subsequently verified by genotyping using PCR and confirmatory DNA sequencing. Confirmed F0 founders were subsequently crossed with *C57BL/6JGpt* mice to generate heterozygous offspring constituting the F1 generation. Homozygous *Cbx2* ^flox/flox^ mice (F2 generation) were obtained through intercrossing and confirmed by PCR genotyping. To generate *Cbx2^LKO^*, *Cbx2* ^flox/flox^ mice were bred with Alb‐Cre mice, while *Cbx2* ^flox/flox^ were used as controls.

### 2.4 Hepatic Steatosis Cell Model

Cellular steatosis was induced in vitro by treating cells with either 0.2 mM palmitic acid (PA) and 0.1 mM oleic acid (OA), or with 30 mM glucose (HGlu) and 100 nM insulin (HIns). In addition, we stimulated inflammatory response by treating the cells with lipopolysaccharide (LPS, 10 µM) for 6 h. For fatty acid metabolism and inflammation assays, cells were transduced with adenoviral vectors for CBX2 overexpression (24 h) or knockdown (48 h) and subsequently exposed to PA/OA for 12 h, with control cells receiving an equivalent volume of 10% bovine serum albumin solution.

Hepatocytes were cultured without serum for 6 h after adenovirus infection. Then stimulated with insulin for 5 min, followed by immediate collection of total protein for analysis of insulin signalling‐related phosphorylation.

### 2.5 Histology and Immunohistochemistry

For hematoxylin and eosin (H&E) staining, sections were incubated in hematoxylin solution for 10–12 min, thoroughly washed under running water, briefly counterstained with eosin for 10s, and subsequently dehydrated and air‐dried. For immunohistochemical staining, after antigen retrieval and blocking endogenous peroxidase, goat serum blocking was performed, subsequently incubated with F4/80 primary antibody. On the following day, sections were exposed to the appropriate secondary antibody, and immunoreactivity was visualized using DAB. All stained sections were visualized using a Leica bright‐field microscope.

### 2.6 Oil Red O Staining

Hepatocytes or frozen liver sections were fixed in 60% isopropanol, stained with 60% oil red solution for 10–15 min.

### 2.7 Quantification of Tissue and Cellular Triglyceride or Cholesterol Levels

Hepatocytes or quantified liver tissue samples should be processed and tested according to the instructions provided in the TG or total cholesterol (TC) assay kit.

### 2.8 Glucose and Insulin Tolerance Tests

Glucose tolerance test (GTT, intraperitoneal D‐glucose injection, 0.75 g kg^−1^ body weight), insulin tolerance test (ITT, intraperitoneal insulin injection, 1.0 IU kg^−1^ body weight), subsequently measure blood glucose at 0, 15, 30, 45, 60, 90, and 120 min.

### 2.9 Calcium Content Determination

Intracellular Ca^2+^ levels in primary mouse hepatocytes were determined using a commercial colorimetric calcium assay kit (575 nm) and Fluo‐4 AM.

### 2.10 Extraction of Histones

After treating primary mouse hepatocytes with Ad‐*Gfp* and Ad‐*Cbx2*, extract histones according to the kit instructions.

### 2.11 Serum Biochemical Analyses

Serum levels of liver injury and inflammation‐related markers were measured using an automated biochemical analyzer (Monach).

### 2.12 RNA Sequencing

RNA sequencing and subsequent bioinformatic analyses were performed by BGI Genomics (Shenzhen, China). After extracting total RNA, polyadenylated transcripts were captured using oligo(dT)‐conjugated magnetic beads and subsequently fragmented. First‐strand complementary DNA was synthesized using random primers, followed by generation of the second‐strand cDNA. Sequencing libraries were generated following sequential end repair and adenylation, adaptor ligation, fragment size selection, PCR enrichment, and final purification. Following quality assessment, libraries were combined according to effective molarity and depth specifications and subsequently sequenced using a high‐throughput Illumina platform. The transcriptomic datasets generated in this work have been submitted to the NCBI Sequence Read Archive, where they are available under the accession identifiers PRJNA1345537 and PRJNA1345185.

### 2.13 Real‐Time Quantitative PCR (RT‐qPCR) and Western Blot Analysis

RT‐qPCR and western blot were performed according to our group's previous reports [20]. Primers and primary antibodies relevant to this study are provided in Tables S1 and S2.

### 2.14 ChIP‐qPCR

Chromatin immunoprecipitation followed by quantitative PCR (ChIP‐qPCR) assays for verifying the binding of H3K4me3 to the *Orai1* promoter region, was supported by Tsingke Biotechnology Co., Ltd. (China).

### 2.15 Statistical Analysis

Results are expressed as mean ± standard deviation (SD). All statistical analyses were conducted using GraphPad Prism 10. Differences between two groups were evaluated using unpaired, two‐tailed Student's t tests, while comparisons involving multiple groups were performed by two‐way analysis of variance (ANOVA). Statistical significance was defined as *p* < 0.05 (*#* or *p* < 0.05; *##* or *p < 0.01; *###* or **p < 0.001).

## 3 Results

### 3.1 CBX2 is Selectively Reduced in Hepatocytes in Human MASLD and Murine Models

As an initial step toward elucidating the contribution of CBX2 to MASLD, we first analyzed publicly available liver transcriptomic datasets (GSE165855 and GSE35961) derived from mouse models of MASLD. Differential expression analysis revealed a marked reduction in *Cbx2* transcript abundance in MASLD liver tissues compared to healthy controls (Figure 1A). Examination of *Cbx2* expression across normal mouse tissues indicated that the liver exhibits the highest basal expression (Figure 1B), suggesting a hepatic‐enriched function. Consistently, hepatic CBX2 expression was markedly reduced in multiple MASLD mouse models, including *db/db* (diabetic), *ob/ob* (obese), HFD‐induced MASLD, and HFHC‐induced steatohepatitis models (Figure 1C–F). In vitro, CBX2 expression was also significantly decreased in primary mouse hepatocytes treated with HGlu + HIns or PA/OA (Figure 1G,H). Importantly, immunohistochemical analysis of human liver biopsies demonstrated substantially lower CBX2 protein levels in patients with MASLD than in healthy controls (Figure 1I).

**FIGURE 1:**
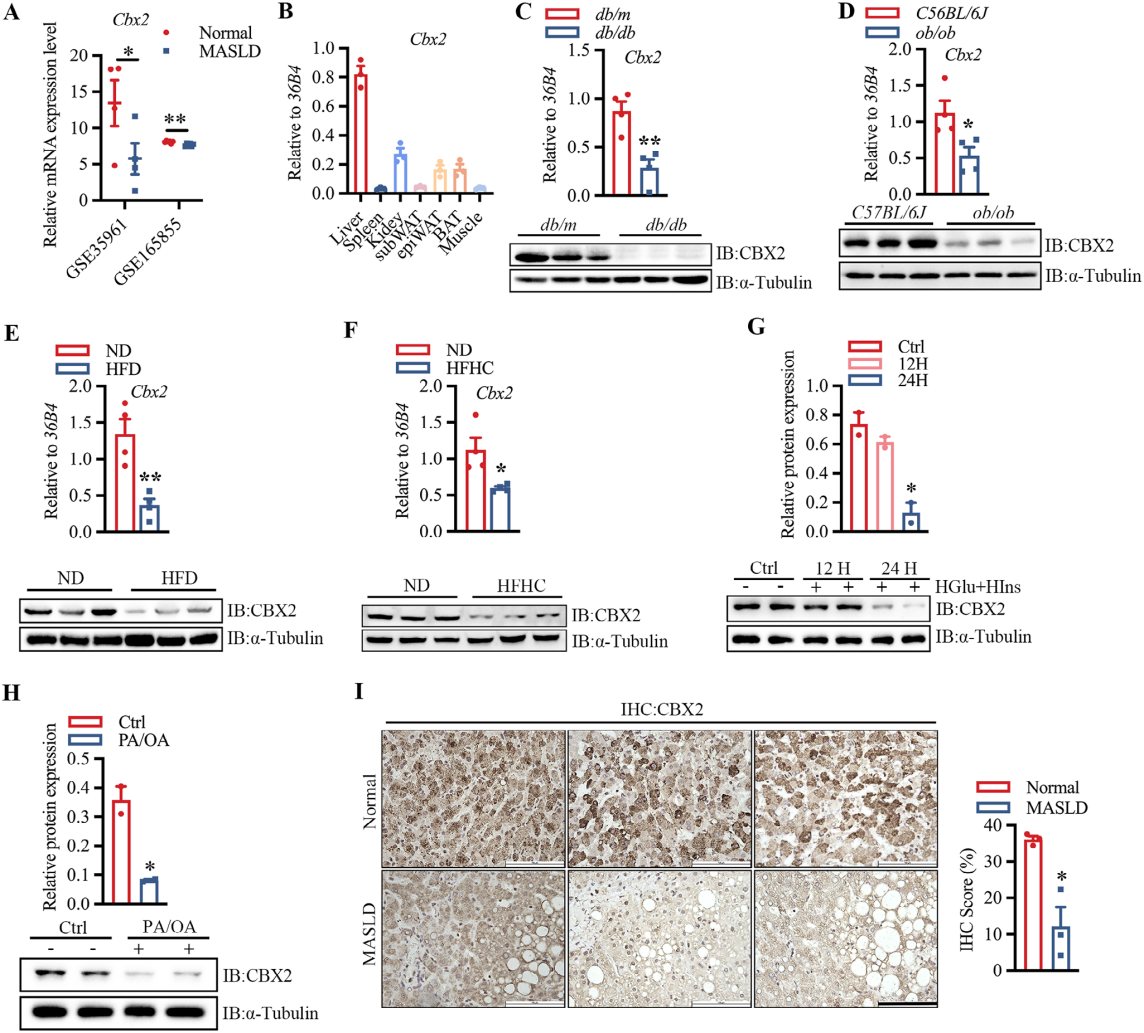
CBX2 expression is decreased in patients and animal models with MASLD. (A) Analysis of liver transcriptome datasets from mouse models of MASLD revealed a significant reduction in *Cbx2* mRNA expression. Data were derived from mice fed a methionine‐ and choline‐deficient (MCD) diet for 8 weeks (GSE35961) and mice fed a high‐fat diet (HFD) for 4 months (GSE165855) (Mean ± SD; *n* = 4–6). (B) *Cbx2* mRNA levels across various tissues in *C57BL/6J* mice (*n* = 6). (C–F) Quantitative RT‐PCR and western blot analyses showing decreased CBX2 expression in the livers of *db/db* mice (C), *ob/ob* mice (D), HFD‐induced obese mice (E), and HFHC‐induced chronic inflammatory mice (F) (*n* = 6 per group). (G, H) Reduced CBX2 expression in hepatocytes following treatment with palmitic acid/oleic acid (PA/OA) or high glucose plus high insulin (HGlu + HIns), mimicking hepatic steatosis conditions. (I) Representative immunohistochemical staining showing diminished CBX2 protein levels in liver tissues from patients with MASLD compared with healthy controls. Data are presented as mean ± SD. *Indicates statistical significance compared to control, *p<0.05, **p<0.01, ***p<0.001.

Collectively, these findings identify CBX2 as a hepatocytes‐enriched factor whose expression is consistently downregulated under steatotic and metabolic stress conditions, suggesting a potential role in MASLD pathogenesis.

### 3.2 Restoring Hepatic CBX2 Improves Systemic Glucose Homeostasis in HFD‐Induced Obesity

Given the consistent downregulation of CBX2 in both murine and human MASLD, we next examined whether restoring its expression could counteract metabolic dysfunction. We employed an adeno‐associated virus system to specifically overexpress CBX2 in the liver of HFD‐fed mice (Figure 2A–C). Targeted upregulation CBX2 levels in the liver led to a significant suppression of weight gain under obese conditions, independent of changes in food consumption (Figure 2D–F). Notably, mice overexpressing CBX2 exhibited substantially lower fasting blood glucose levels relative to AAV‐*Gfp* control mice (Figure 2G). Although the expression of gluconeogenic genes, including phosphoenolpyruvate carboxykinase 1 and glucose‐6‐phosphatase, was not significantly altered (Figure S1), both GTT and ITT demonstrated that CBX2 overexpression alleviated HFD‐induced glucose intolerance and insulin resistance (Figure 2H,I). Western blot analysis further showed enhanced insulin‐stimulated phosphorylation of protein kinase B (AKT) and glycogen synthase kinase‐3β (GSK‐3β) in the livers of AAV‐*Cbx2* mice (Figure 2J,K).

**FIGURE 2:**
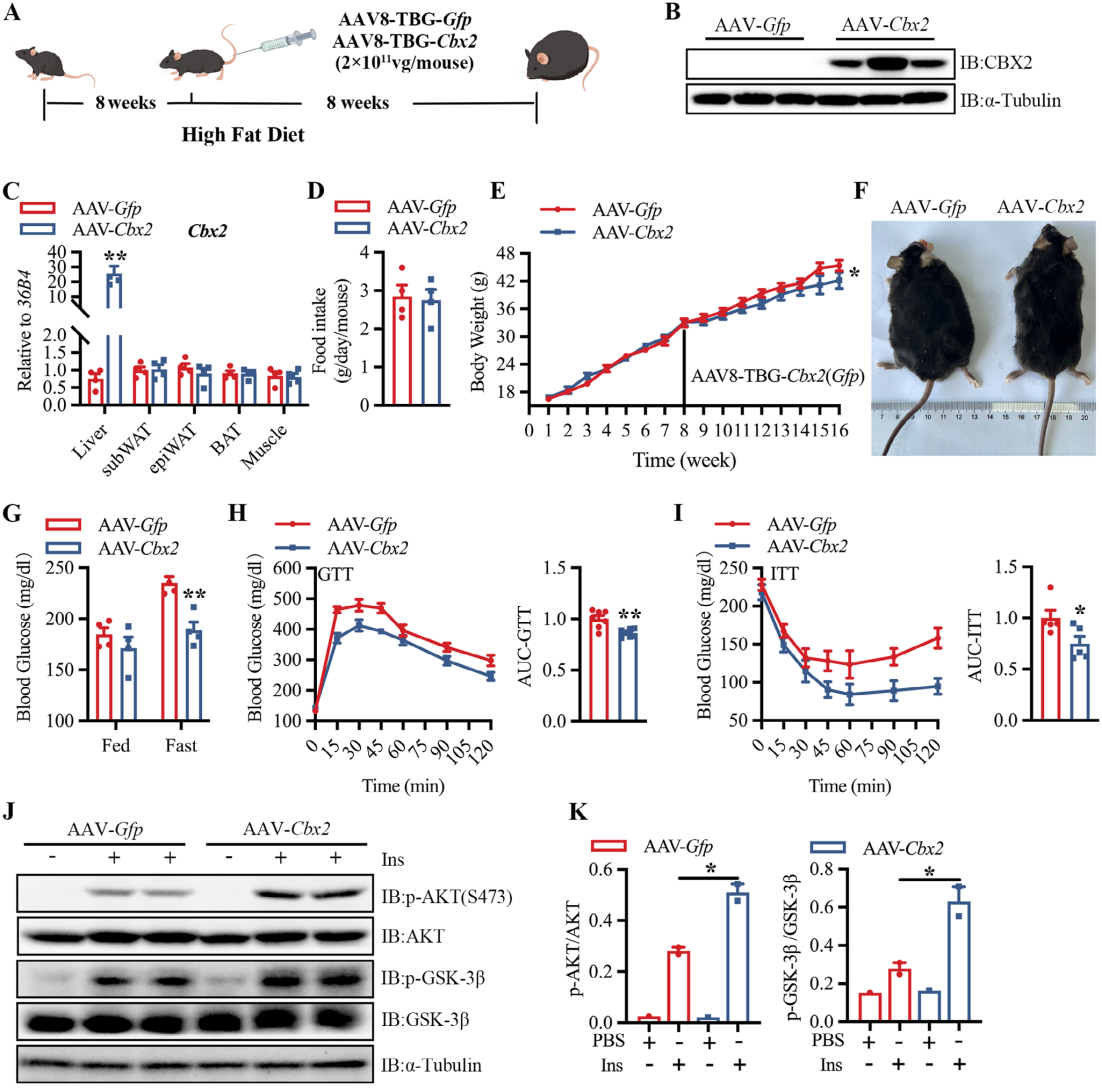
Hepatic overexpression of CBX2 reduces body weight and improves insulin resistance in HFD‐induced obese mice. (A) Schematic representation of the experimental design showing HFD feeding and recombinant adeno‐associated virus (AAV) administration via tail vein injection. (B,C) Validation of hepatic CBX2 overexpression by western blot (B) and RT‐qPCR (C) analyses in HFD‐induced obese mice (*n* = 8 per group). (D) Food intake in AAV‐*Gfp* and AAV‐*Cbx2* groups. (E) Weekly body weight changes in HFD‐fed mice following AAV‐*Gfp* or AAV‐*Cbx2* administration. (F) Representative images showing the body morphology of AAV‐*Gfp* and AAV‐*Cbx2* mice. (G) Blood glucose levels measured under fed and 6 h fasting conditions in both groups. (H,I) GTT and ITT showing improved glucose and insulin sensitivity in AAV‐*Cbx2* mice compared with AAV‐*Gfp* controls. (J) Western blot analysis of insulin‐stimulated phosphorylation of AKT and GSK‐3β in liver tissues. (K) Quantification of AKT and GSK‐3β phosphorylation levels in (J). Data are presented as mean ± SD. *Indicates statistical significance compared to AAV‐*Gfp*, *p<0.05, **p<0.01, ***p<0.001.

Collectively, these findings support a role for hepatic CBX2 in maintaining glucose homeostasis and insulin sensitivity, mitigating the metabolic disturbances caused by HFD‐induced obesity.

### 3.3 CBX2 Curbs Diet‐Induced Hepatic Steatosis and Inflammatory Activation Under HFD

Encouraged by the metabolic improvements observed upon CBX2 restoration, we further explored whether CBX2 overexpression could also alleviate hepatic steatosis and inflammation‐key pathological features of MASLD. As shown in Figure 3A, livers from HFD‐fed control mice appeared enlarged and yellowish, indicative of severe lipid accumulation, whereas AAV‐*Cbx2‐*treated livers exhibited a near‐normal appearance. CBX2 overexpression significantly decreased the liver/body weight (Figure 3B). Histological analyses demonstrated that hepatic CBX2 overexpression substantially attenuated HFD‐induced steatosis and lipid droplet accumulation (Figure 3C). Similarly, hepatic TG and TC contents were reduced upon CBX2 overexpression (Figure 3D,E). Biochemical assays further showed that serum TG and TC levels were significantly lower in AAV‐*Cbx2* mice compared with AAV‐*Gfp* controls, while FFA remained unchanged (Figure 3F–H). In addition, serum levels of ALT, AST, and tumor necrosis factor‐α (TNFα) were significantly decreased in CBX2‐overexpressing mice (Figure 3I–K), suggesting improved liver function and reduced inflammation. Immunostaining for F4/80 confirmed that hepatic macrophage infiltration was markedly diminished following CBX2 overexpression (Figure 3L).

**FIGURE 3:**
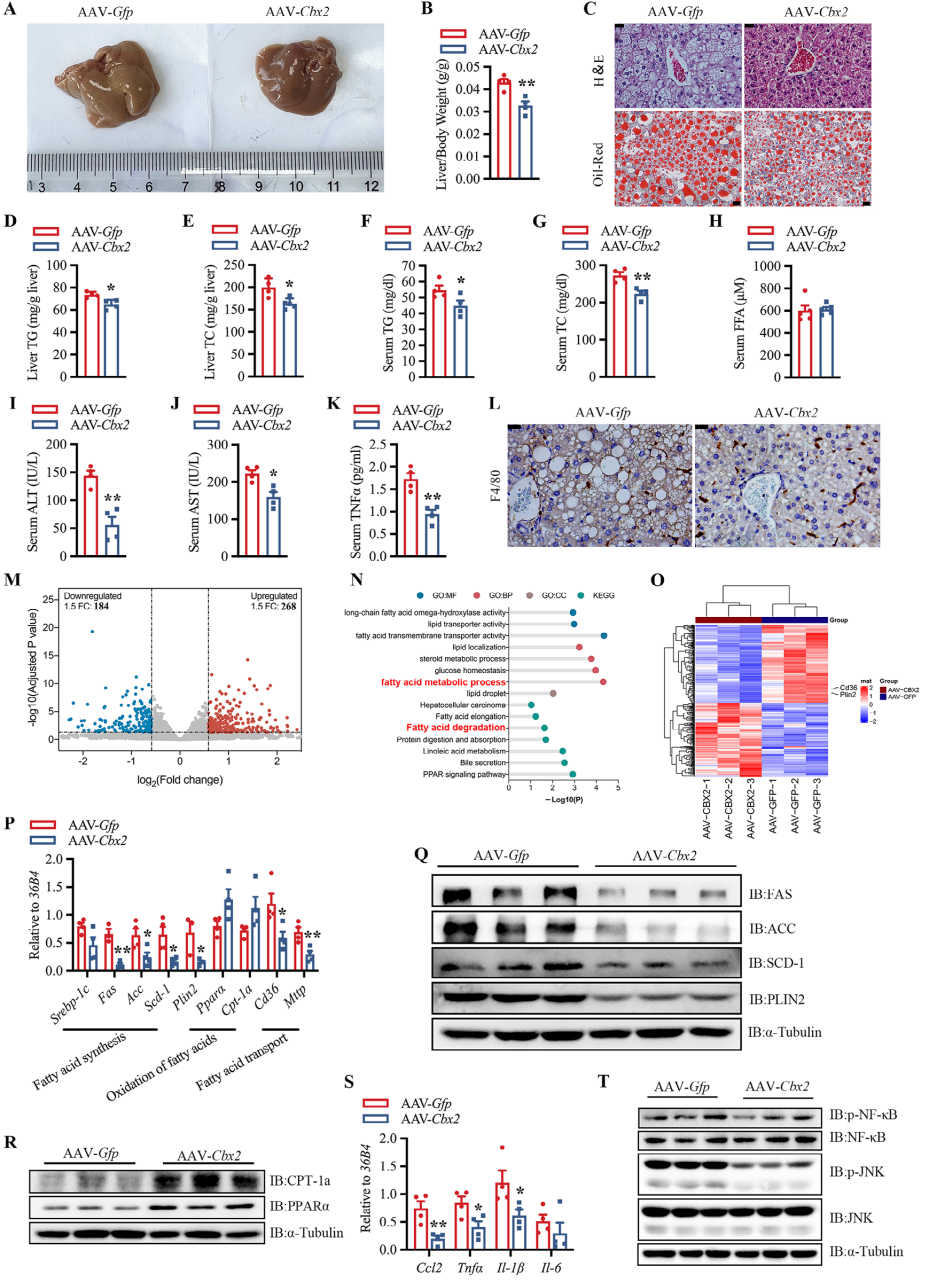
Hepatic CBX2 overexpression alleviates HFD‐induced hepatic steatosis. (A) Representative liver morphology of AAV‐*Gfp* and AAV‐*Cbx2* mice following HFD feeding. (B) Liver to body weight ratios in the two groups. (C) Representative H&E and Oil Red O staining of liver biopsies (×400; scale bars, 20 µm). (D,E) Liver TG and TC contents. (F‐H) Serum TG, TC, and FFA levels. (I–K) Serum ALT, AST, and TNFα levels. (L) Immunohistochemical staining of F4/80 in liver sections (×400, scale bars, 20 µm) from AAV‐*Gfp* and AAV‐*Cbx2* mice. (M) Scatter plot of differentially expressed genes identified by RNA‐seq in liver tissues from AAV‐*Gfp* and AAV‐*Cbx2* mice. (N) Gene Ontology (GO) and KEGG pathway enrichment analyses of metabolic genes identified by RNA‐seq. (O) Heatmap showing expression profiles of genes involved in hepatic lipid metabolism based on RNA‐seq. (P) RT‐qPCR validation of genes associated with fatty acid synthesis, oxidation, and transport in livers of HFD‐fed mice. (Q,R) Western blot analyses of protein levels associated with fatty acid synthesis (Q) and fatty acid oxidation (R). (S) RT‐qPCR analysis of inflammation‐related genes expression in liver tissues. (T) Western blot analysis of total and phosphorylated JNK and NF‐κB proteins in the livers of AAV‐*Gfp* and AAV‐*Cbx2* mice. Data are presented as mean ± SD. *Indicates statistical significance compared to AAV‐*Gfp*, *p<0.05, **p<0.01, ***p<0.001.

To explore the molecular basis of CBX2‐driven hepatoprotection, RNA‐seq was performed on liver tissues from HFD mice expressing CBX2 or control vectors. Transcriptomic profiling revealed a total of 452 genes exhibiting differential expression, including 286 transcripts that were increased and 184 that were decreased (Figure 3M). Gene ontology and pathway enrichment analyses revealed that CBX2 overexpression significantly enhanced pathways related to fatty acid transmembrane transport, fatty acid metabolism, and fatty acid degradation (Figure 3N). Heatmap analysis of lipid metabolism‐related genes showed that CBX2 overexpression suppressed the expression of *Cd36* (a major fatty acid transporter) and *Plin2* (a lipid droplet‐associated protein) (Figure 3O). In agreement, CBX2 overexpression suppressed the expression of major DNL genes (FAS, ACC and SCD‐1), as revealed by RT‐qPCR and western blot analyses. (Figure 3P,Q).

Peroxisome proliferator‐activated receptor α (PPARα), a central regulator of mitochondrial fatty acid oxidation, promotes the expression of fatty acid oxidizing enzymes including carnitine palmitoyl transferase‐1a (CPT‐1a) [21]. Hepatic CBX2 overexpression markedly increased the protein levels of PPARα and its downstream effector CPT‐1a, as shown by western blot (Figure 3R). In parallel, RT‐qRCR and western blot assays revealed that CBX2 overexpression suppressed proinflammatory cytokines (*Ccl2*, *Tnfα*, *Il‐1β*, and *Il‐6*) and inhibited the phosphorylation of nuclear factor κB (NF‐κB) and c‐Jun N‐terminal kinase (JNK), two key inflammatory signaling mediators (Figure 3S,T). Of note, hepatic CBX2 overexpression did not produce appreciable metabolic effects under normal chow conditions (Figure S2). Collectively, these findings demonstrate that hepatic CBX2 overexpression robustly mitigates HFD‐induced steatosis, dyslipidemia, and inflammation by promoting fatty acid oxidation and repressing lipogenesis and inflammatory signaling.

As T2DM represents a major risk factor for MASLD, and hepatic CBX2 expression is significantly downregulated in *db/db* mice (Figure 1C), we further assessed whether CBX2 restoration could ameliorate metabolic abnormalities in this model. Liver‐specific CBX2 overexpression significantly ameliorated glucose intolerance and insulin resistance in *db/db* mice, without significantly altering body weight or fasting blood glucose levels (Figure S3). However, only partial improvements in hepatic steatosis and inflammatory responses were observed (Figure S4), suggesting that CBX2's protective effects may be limited under severe diabetic conditions.

### 3.4 CBX2 Protects Against HFHC‐Driven Steatohepatitis and Dyslipidemia

To explore how CBX2 influences the progression of MASH, we employed an HFHC diet to induce chronic steatohepatitis in mice. Long‐term HFHC feeding has been reported to cause obesity, dyslipidemia, hepatomegaly, MASH, and mild fibrosis [22]. After 8 weeks of HFHC feeding, mice were injected of AAV‐*Cbx2* or AAV‐*Gfp* and maintained on the same diet for 8 weeks (Figure 4A). Efficient hepatic overexpression of CBX2 was confirmed (Figure S5).

**FIGURE 4:**
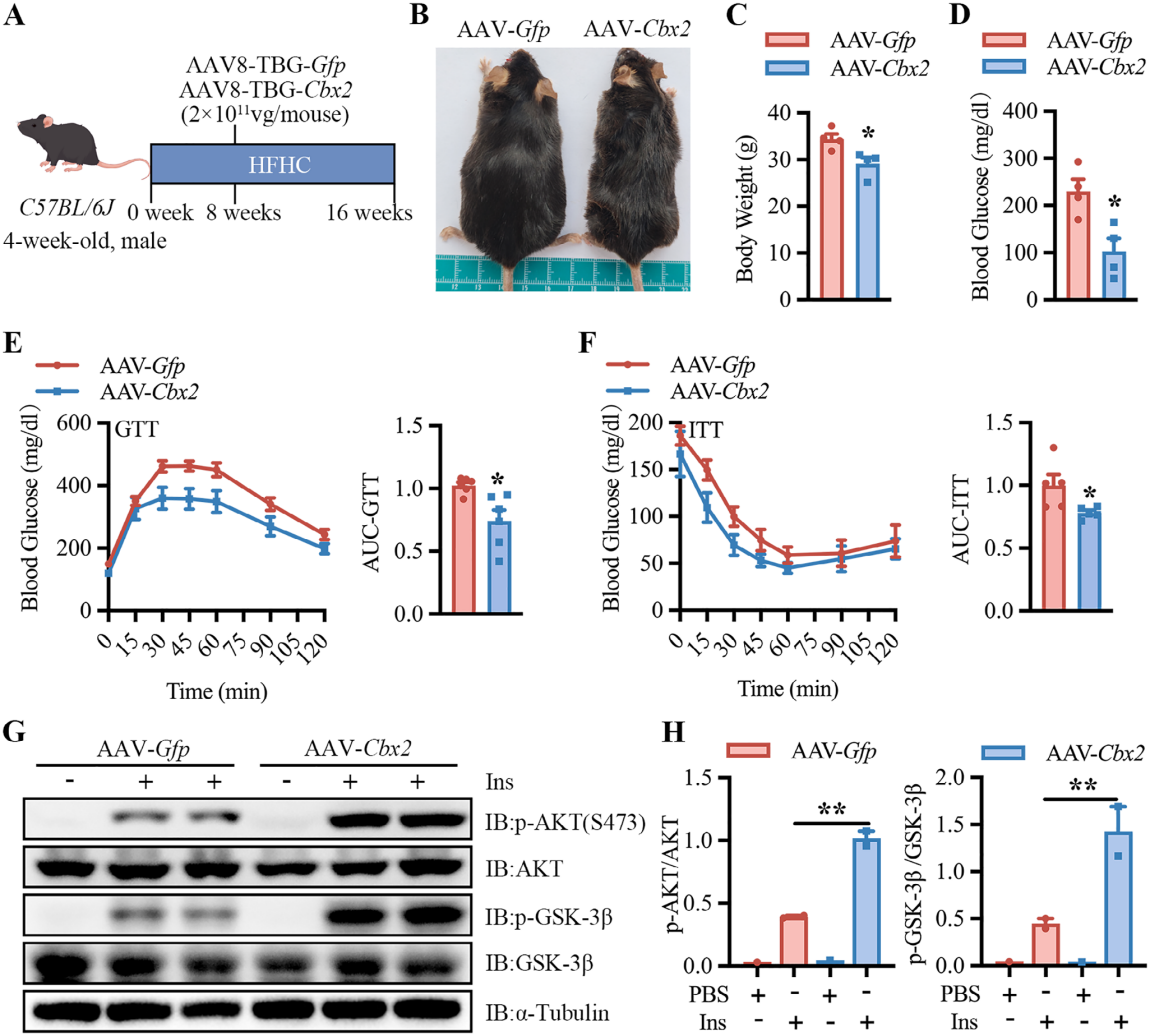
Hepatic CBX2 overexpression improves insulin sensitivity in HFHC‐induced chronic inflammation mice. (A) Schematic representation of the experimental design showing HFHC diet feeding and recombinant adeno‐associated virus (AAV) administration via tail vein injection. (B) Representative gross morphology of AAV‐*Gfp* and AAV‐*Cbx2* mice. (C,D) Overexpression of CBX2 significantly reduced body weight (C) and blood glucose levels (D) in HFHC‐induced chronic inflammation mice. (E,F) GTT and ITT showing improved glucose and insulin sensitivity in AAV‐*Cbx2* mice compared with AAV‐*Gfp* controls. (G) Western blot analysis of insulin‐stimulated phosphorylation of AKT and GSK‐3β in liver tissues from AAV‐*Gfp* and AAV‐*Cbx2* mice. (H) Quantification of AKT and GSK‐3β phosphorylation levels in (G). Data are presented as mean ± SD. *Indicates statistical significance compared to AAV‐*Gfp*, *p<0.05, **p<0.01, ***p<0.001.

Relative to controls, hepatic CBX2 overexpression led to a pronounced reduction in body weight and a significant decrease in glucose levels in HFHC‐fed mice (Figure 4B–D). GTT and ITT further confirmed that CBX2 improved both glucose tolerance and insulin sensitivity (Figure 4E,F), accompanied by increased phosphorylation of AKT and GSK‐3β in the liver (Figure 4G,H), indicating enhanced insulin signaling.

Consistent with findings from HFD‐fed obese mice, livers of HFHC‐fed mice with overexpressing CBX2 appeared reddish and smooth with a lower liver/body weight ratio (Figure 5A,B). H&E and Oil Red O staining revealed that CBX2 overexpression significantly reduced hepatic steatosis and lipid deposition (Figure 5C). Biochemical measurements showed that TG, TC, and FFA levels were significantly lowered, while hepatic TG content was also markedly reduced (Figure 5D–H).

**FIGURE 5:**
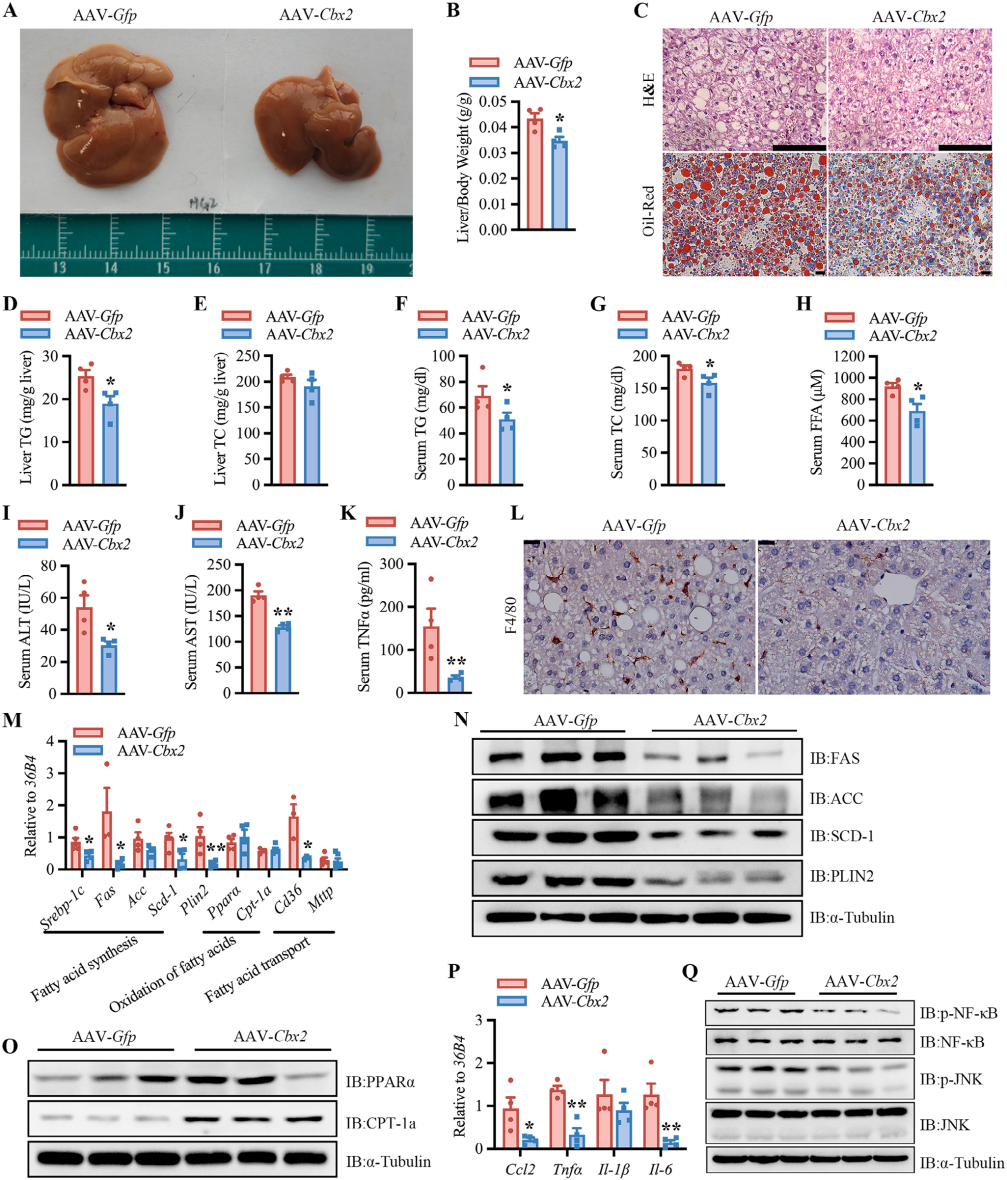
Hepatic CBX2 overexpression ameliorates lipid accumulation and inflammation in HFHC‐induced chronic inflammation mice. (A) Representative liver morphology of AAV‐*Gfp* and AAV‐*Cbx2* mice. (B) CBX2 overexpression significantly reduced the liver‐to‐body weight ratio in HFHC‐induced chronic inflammation mice. (C) Representative H&E and Oil red O staining of liver sections (H&E: ×400, scale bars, 100 µm; Oil Red O: ×400, scale bars, 20 µm). (D,E) Hepatic TG and TC contents. (F–H) Serum TG, TC, and FFA levels following CBX2 overexpression. (I–K) *S*erum ALT, AST, and TNFα levels. (L) Immunohistochemical staining of F4/80 showing reduced macrophage infiltration in the livers of AAV‐*Cbx2* mice (×400; scale bars, 20 µm). (M–O) RT‐qPCR and western blot analyses showing that CBX2 overexpression suppressed the expression of fatty acid synthesis genes and enhanced the expression of fatty acid oxidation‐related genes. (P) RT‐qPCR analysis showing that CBX2 overexpression downregulated inflammation‐related gene expression. (Q) Western blot analysis showing that CBX2 overexpression markedly reduced the phosphorylation levels of NF‐κB and JNK in liver tissues. Data are presented as mean ± SD. *Indicates statistical significance compared to AAV‐*Gfp*, *p<0.05, **p<0.01, ***p<0.001.

Furthermore, serum ALT, AST, and TNFα levels were significantly lower in AAV‐*Cbx2* treatment mice (Figure 5I–K), indicating alleviated liver injury and inflammation. Immunostaining for F4/80 revealed that CBX2 overexpression markedly reduced macrophage infiltration in HFHC‐fed livers (Figure 5L). RT‐qPCR and western blot analyses demonstrated that CBX2 overexpression suppressed lipogenic gene expression (e.g., FAS, SCD‐1, ACC, PLIN2) while upregulating fatty acid oxidation genes (e.g., PPARα, CPT‐1a) (Figure 5M–O). In parallel, CBX2 overexpression significantly downregulated inflammatory mediators (*Ccl2*, *Tnfα*, *Il‐6*) and inhibited NF‐κB and JNK phosphorylation (Figure 5P,Q).

Collectively, these results demonstrate that hepatic CBX2 overexpression ameliorates HFHC diet‐induced hepatic steatosis and inflammation by improving metabolic homeostasis, reducing lipid accumulation, and suppressing inflammatory signaling.

### 3.5 Hepatic CBX2 Loss Aggravates Steatosis, Insulin Resistance, and Inflammation in Obesity

To probe the contribution of hepatic CBX2 to obesity‐related metabolic impairments, HFD‐induced obese mice were administered AAV‐sh*Cbx2* to selectively silence *Cbx2* in the liver (Figure S6A,B). Loss of hepatic CBX2 aggravated HFD‐induced metabolic dysfunction, manifesting as increased body weight and elevated blood glucose (Figure S6C,D). GTT and ITT further demonstrated that CBX2 deficiency markedly impaired glucose homeostasis and insulin responsiveness relative to controls (Figure S6E,F). Consistently, insulin‐stimulated phosphorylation of hepatic AKT and GSK‐3β was markedly reduced in CBX2‐deficient mice (Figure S6G), indicating compromised insulin signaling.

Histological and biochemical analyses revealed that CBX2 knockdown intensified hepatic steatosis and increased the liver/body weight (Figure S7A–C). Hepatic TG and TC levels, along with serum TG and FFA contents, were significantly elevated in CBX2‐deficient mice, whereas serum TC levels remained unaffected (Figure S7D–H). F4/80 immunostaining indicated increased macrophage infiltration in the liver, accompanied by elevated serum ALT and TNFα levels (Figure S7I–L), suggesting exacerbated hepatic inflammation.

At the molecular level, RT‐qPCR and western blot analyses showed that CBX2 knockdown upregulated the expression of lipogenic genes (FAS, ACC) and lipid droplet‐associated PLIN2, while downregulating PPARα, CPT‐1a (Figure S7M,N). Moreover, phosphorylation levels of key inflammatory mediators NF‐κB and JNK were increased upon CBX2 knockdown (Figure S7O–Q). Collectively, these findings demonstrate that hepatic CBX2 deficiency aggravates lipid accumulation, impairs insulin signaling, and promotes inflammation in HFD‐fed mice.

### 3.6 Re‐Expressing CBX2 Rescues Metabolic Defects in Hepatocyte‐Specific Knockouts

Hepatocyte‐specific Cbx2‐knockout mice (*Cbx2^LKO^*) were generated, with *Cbx2^flox/flox^* littermates serving as controls, to further explore CBX2's role in liver metabolism (Figure S8A–C). *Cbx2^LKO^* mice received a single tail‐vein injection of AAV‐*Cbx2* after 8 weeks of HFD, restoring hepatic CBX2 expression, and were maintained on HFD for a further 8 weeks (Figure 6A). RT‐qPCR and western blot results confirmed efficient deletion of *Cbx2* in *Cbx2^LKO^* livers and successful re‐expression upon AAV‐*Cbx2* treatment (Figure S8D,E).

**FIGURE 6:**
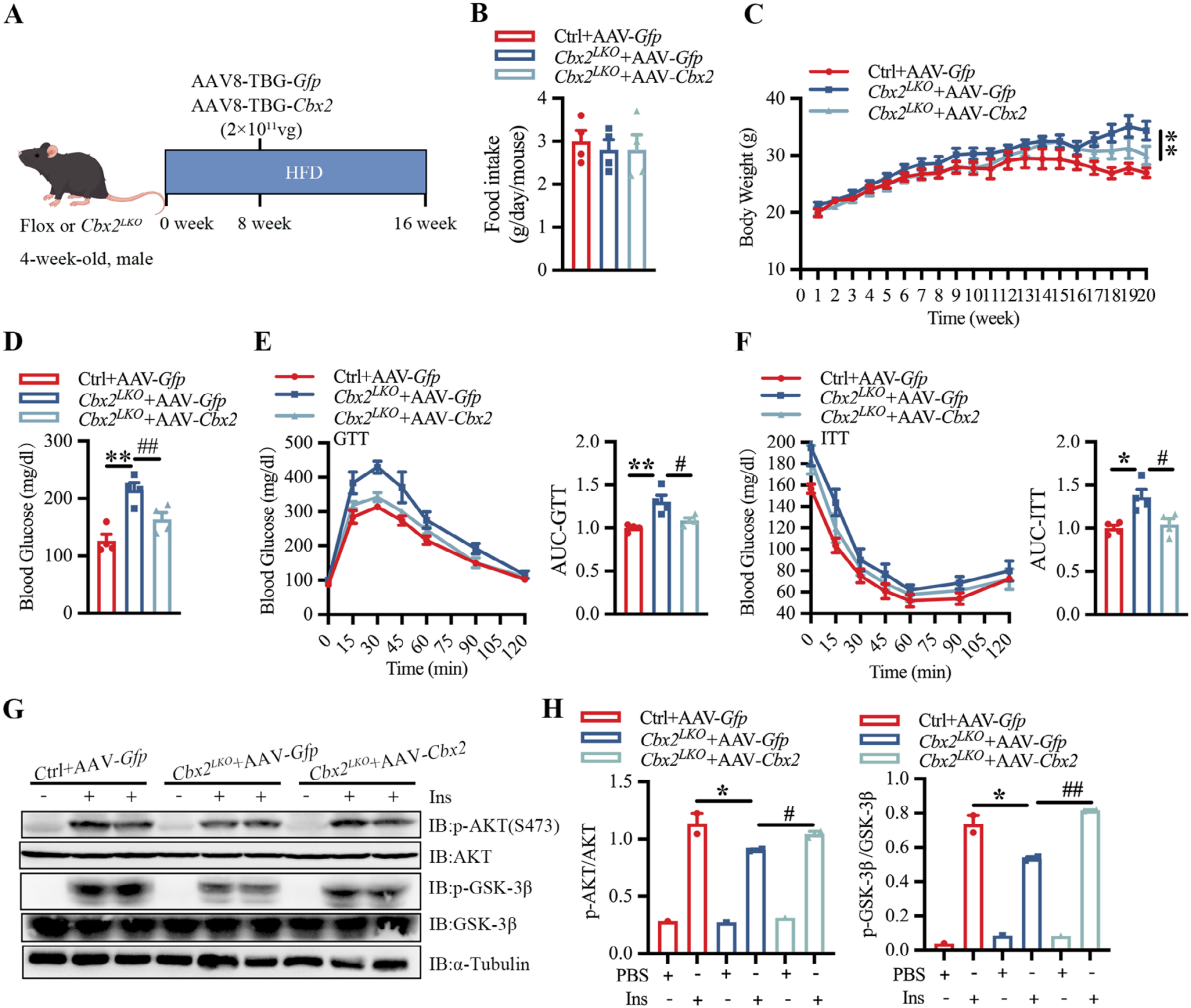
Restoration of hepatic CBX2 expression rescues exacerbated insulin resistance in *Cbx2^LKO^* mice. (A) Experimental design: *Cbx2^Flox^* mice and CBX2 liver‐specific knockout (*Cbx2^LKO^*) mice (4 weeks old) were fed an HFD for 8 weeks, followed by intravenous tail vein injection of AAV8‐TBG‐*Gfp* or AAV8‐TBG‐*Cbx2*, and maintained on HFD for an additional 8 weeks (*n* = 8 per group). (B) Food intake across experimental groups. (C) Sequential body weight changes during the experimental period. (D) Blood glucose levels in each group. (E,F) GTT and ITT demonstrating that hepatic CBX2 restoration ameliorated glucose intolerance and insulin resistance in *Cbx2^LKO^* mice. (G) Western blot analysis insulin‐stimulated phosphorylation of AKT and GSK‐3β in liver tissues of each group. (H) Quantification of AKT and GSK‐3β phosphorylation levels shown in (G). Data are presented as mean ± SD. *Indicates statistical significance compared to Ctrl + AAV‐*Gfp*, ^#^Indicates statistical significance compared to *Cbx2^LKO^* +AAV‐*Gfp*, * ^or #^ *p*<0.05, ** ^or ##^ *p*<0.01, *** ^or ###^ *p*<0.001.

Compared with *Cbx2^flox/flox^*, *Cbx2^LKO^* mice exhibited accelerated body‐weight gain under HFD conditions, without changes in food intake, whereas hepatic CBX2 re‐expression significantly mitigated this increase (Figure 6B,C). AAV‐*Cbx2* treatment also normalized fasting blood glucose levels in *Cbx2^LKO^* mice (Figure 6D). GTT and ITT revealed that CBX2 deficiency impaired glucose tolerance and insulin sensitivity, both of which were substantially restored by CBX2 overexpression (Figure 6E,F). Furthermore, insulin‐induced phosphorylation of AKT and GSK‐3β was reduced in *Cbx2^LKO^* livers, as shown by western blot but partially rescued by AAV‐*Cbx2* re‐expression (Figure 6G,H).

Together, these results establish CBX2 as a crucial regulator of hepatic lipid and glucose homeostasis, where loss of CBX2 exacerbates, and its restoration ameliorates, HFD‐induced metabolic dysfunction.

### 3.7 Restoration of CBX2 Reverses Steatosis and Hepatic Inflammation in *Cbx2^LKO^* Livers

Macroscopic inspection revealed that livers from *Cbx2^LKO^* mice appeared pale and dull following HFD feeding, while AAV*‐Cbx2* delivery restored their normal pink color, suggesting alleviation of hepatic steatosis (Figure 7A). Histopathological assessment using H&E and Oil Red O staining further confirmed that CBX2 deficiency aggravated hepatic lipid accumulation and tissue injury, whereas CBX2 re‐expression markedly attenuated these changes (Figure 7B). The number of F4/80‐positive macrophages, indicative of hepatic inflammation, was also reduced upon CBX2 restoration (Figure 7B). Consistently, AAV‐*Cbx2* treatment normalized the increased liver/body weight in knockout mice (Figure 7C).

**FIGURE 7:**
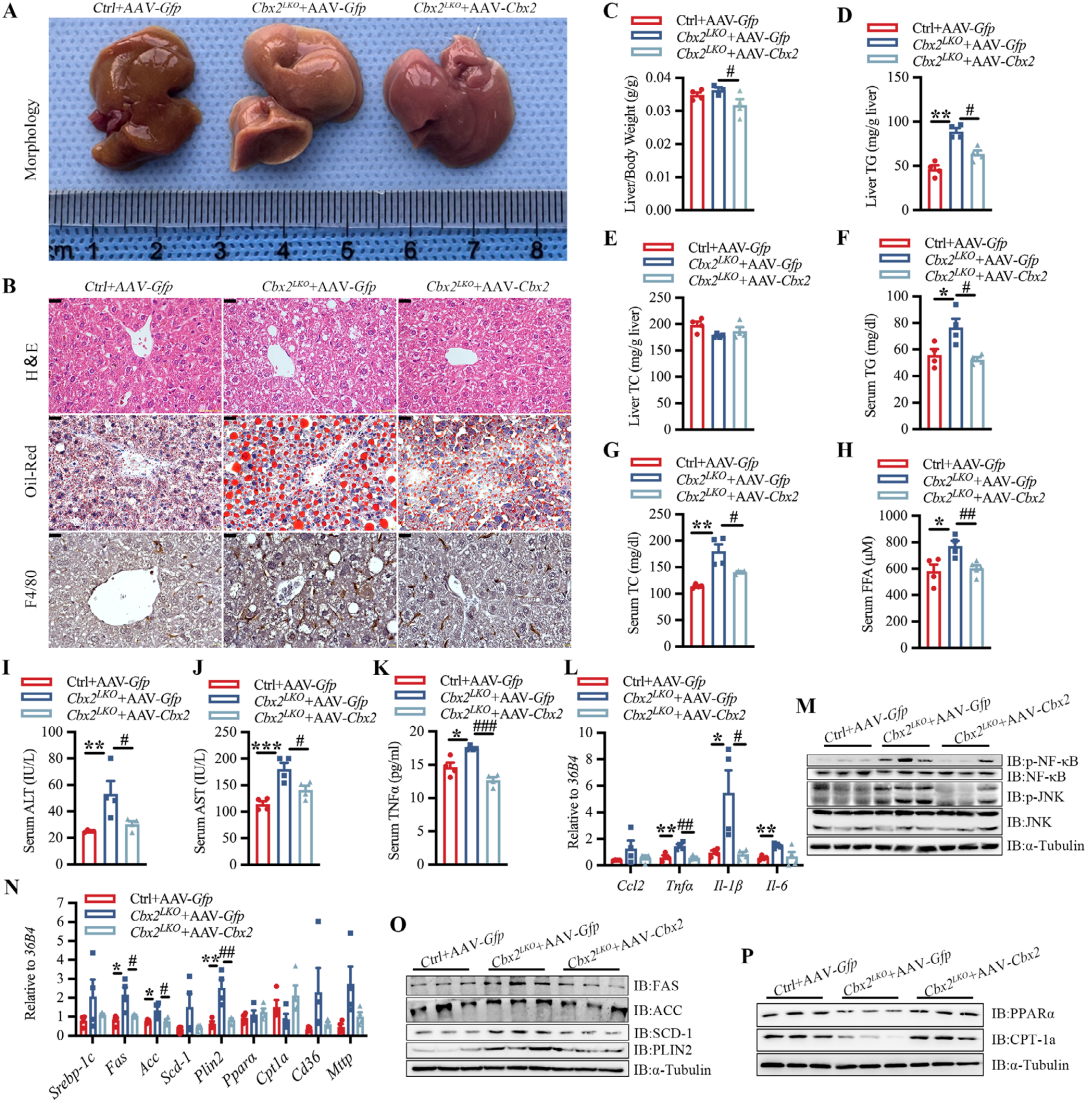
Restoration of hepatic CBX2 expression ameliorates exacerbated MASLD in *Cbx2^LKO^* mice. (A) Representative liver morphology of each experimental group. (B) Representative H&E staining, Oil Red O, and F4/80 immunostaining of liver sections showing hepatic steatosis and macrophage infiltration (×400, scale bars, 20 µm). (C) Liver‐to‐body weight ratios in each group. (D, E) Hepatic TG and TC contents. (F‐H) Serum TG, TC, and FFA levels. (I‐K) Serum levels of ALT, AST, and TNFα. (L) RT‐qPCR analysis of inflammation‐related gene expression in liver tissues. (M) Western blot analysis of total and phosphorylated NF‐κB and JNK in liver tissues. (N–P) RT‐qPCR and western blot analyses showing that CBX2 restoration suppressed fatty acid synthesis‐related genes and enhanced fatty acid oxidation‐related gene expression. Data are presented as mean ± SD. *Indicates statistical significance compared to Ctrl + AAV‐*Gfp*, ^#^Indicates statistical significance compared to *Cbx2^LKO^* +AAV‐*Gfp*, * ^or #^ *p*<0.05, ** ^or ##^ *p*<0.01, *** ^or ###^ *p*<0.001.

Biochemical measurements corroborated these findings: AAV‐*Cbx2* significantly decreased hepatic TG content, as well as serum TG, TC, and FFA levels, without significantly affecting hepatic TC levels (Figure 7D–H). Restoration of CBX2 also lowered serum ALT, AST, and TNFα levels, indicating improved liver function and reduced systemic inflammation (Figure 7I–K).

CBX2 overexpression attenuated the transcriptional activation of proinflammatory genes induced by HFD, as revealed by RT‐qPCR (Figure 7L). In agreement, western blot analysis showed reduced phosphorylation of NF‐κB and JNK, key mediators of inflammatory signaling (Figure 7M). Restoration of hepatic CBX2 via AAV‐*Cbx2* reduced lipogenic gene expression and PLIN2 levels, while promoting the expression of PPARα and CPT‐1a (Figure 7N–P).

Together, these results indicate that restoration of hepatic CBX2 mitigates HFD‐induced steatosis, inflammation, and metabolic dysregulation, highlighting CBX2 as a potential therapeutic target for MASLD.

### 3.8 CBX2 Limits Lipotoxicity and Strengthens Insulin Signaling in Primary Hepatocytes

After primary mouse hepatocytes were infected with adenovirus overexpressing or knocking down CBX2 and exposed to PA/OA to assess CBX2's role in lipid metabolism. Upon CBX2 overexpression (Figure 8A and Figure S9A), Oil Red O staining and biochemical quantification revealed that CBX2 overexpression markedly attenuated PA/OA‐induced lipid droplet formation and significantly reduced intracellular TG levels (Figure 8B,C). Consistently, western blot analysis demonstrated that CBX2 overexpression inhibited the PA/OA‐induced upregulation of lipogenic enzymes (FAS, ACC, and SCD‐1) and the lipid droplet‐associated protein PLIN2 (Figure 8D), while concomitantly enhancing the expression of PPARα and CPT‐1a (Figure 8E).

**FIGURE 8:**
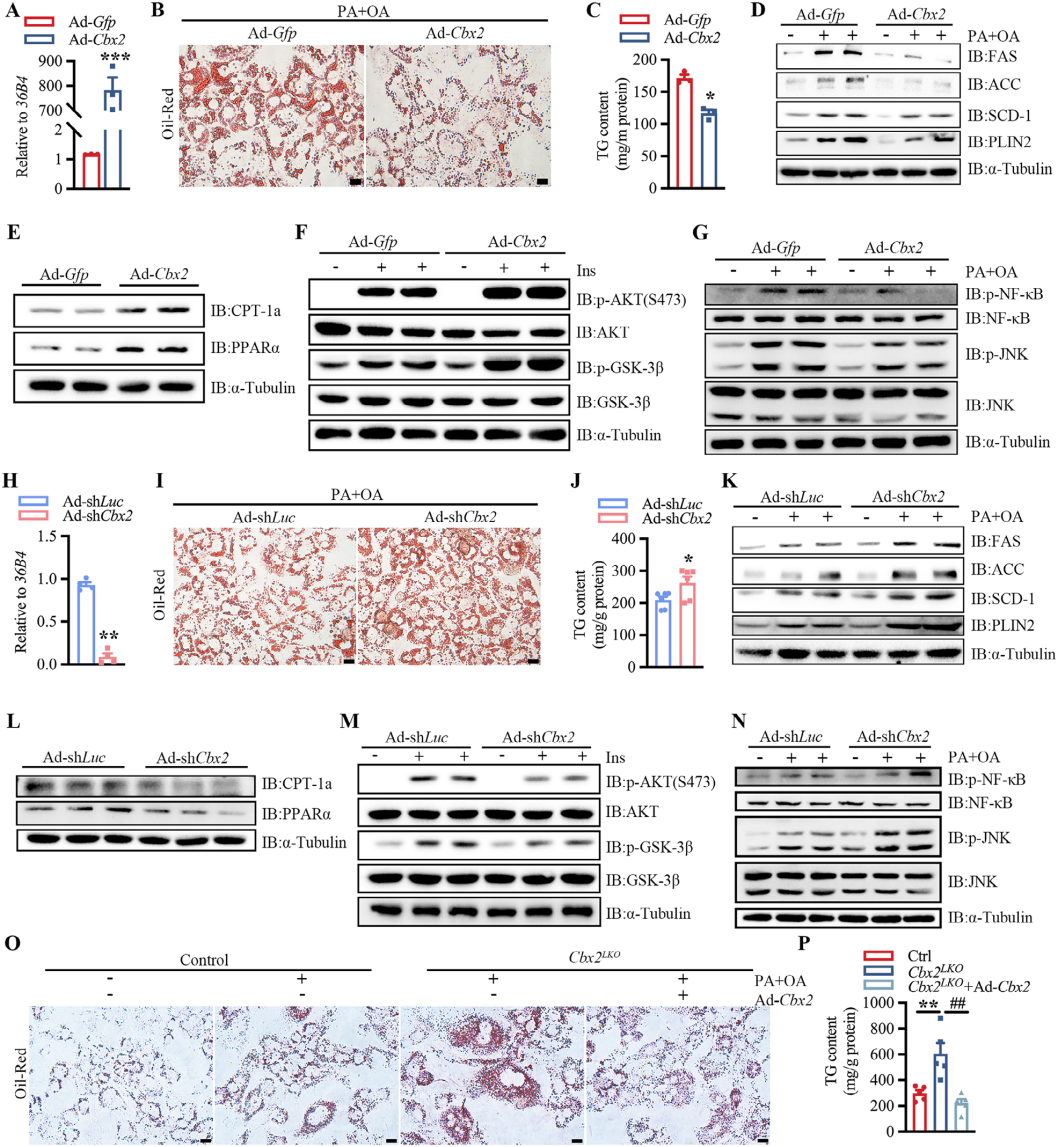
CBX2 regulates fatty acid metabolism, enhances insulin sensitivity, and attenuates inflammation to reduce lipid accumulation in primary mouse hepatocytes. (A) RT‐qPCR analysis showing increased *Cbx2* mRNA levels in primary mouse hepatocytes after 24 h of adenoviral *Cbx2* (Ad‐*Cbx2*) infection. (B) Oil Red O staining showing that CBX2 overexpression suppressed palmitic acid/oleic acid (PA/OA) ‐induced lipid droplet formation (×400; scale bar, 20 µm). (C) CBX2 overexpression reduced PA/OA‐induced TG accumulation in primary hepatocytes. (D, E) RT‐qPCR analysis showing that CBX2 overexpression inhibited PA/OA‐induced fatty acids synthesis gene expression (D) while enhancing fatty acid oxidation‐related gene expression (E). (F) Western blot analysis showing increased phosphorylation of AKT and GSK‐3β following CBX2 overexpression. (G) CBX2 overexpression reduced PA/OA‐induced phosphorylation of NF‐κB and JNK in primary hepatocytes. (H) Efficient knockdown of CBX2 expression in primary hepatocytes using adenoviral shRNA (Ad‐sh*Cbx2)*. (I) Oil Red O staining showing that CBX2 knockdown promoted PA/OA‐induced lipid droplet accumulation (×400; scale bar, 20 µm). (J) CBX2 knockdown increased PA/OA‐induced TG synthesis in mouse primary hepatocytes. (K,L) RT‐qPCR analysis showing that CBX2 knockdown enhanced PA/OA‐induced expression of fatty acid synthesis genes (K) and suppressed fatty acid oxidation‐related genes (L). (M) Western blot analysis showing reduced phosphorylation of AKT and GSK‐3β following CBX2 knockdown. (N) CBX2 knockdown increased PA/OA‐induced phosphorylation of NF‐κB and JNK in primary hepatocytes. (O) Oil Red O staining showing increased lipid droplet accumulation in primary hepatocytes isolated from *Cbx2^LKO^* mice. (P) TG content in primary hepatocytes from *Cbx2^LKO^* mice. Data are presented as mean ± SD. *Indicates statistical significance compared to Ad‐*Gfp* or Ad‐sh*Luc*; *p<0.05, **p<0.01, ***p<0.001. ^#^Indicates statistical significance compared to *Cbx2^LKO^*; ^#^ *p*<0.05, ^##^ *p*<0.01, ^###^ *p*<0.001.

Conversely, CBX2 knockdown aggravated PA/OA‐induced lipid accumulation, as evidenced by increased lipid droplet formation (Figure 8H,I and Figure S9B) and elevated TG levels (Figure 8J). Loss of CBX2 upregulated FAS, ACC, SCD‐1, and PLIN2 (Figure 8K) and suppressed the expression of PPARα and CPT‐1a (Figure 8L), indicating a shift toward lipogenesis.

Moreover, CBX2 overexpression enhanced insulin signaling, as shown by increased phosphorylation of AKT and GSK‐3β, without altering total protein levels (Figure 8F). In contrast, CBX2 depletion markedly reduced phosphorylation of both proteins (Figure 8M). CBX2 also mitigated PA/OA‐induced inflammation, as reflected by reduced phosphorylation of NF‐κB and JNK (Figure 8G), whereas CBX2 silencing promoted their activation (Figure 8N). In addition, we used LPS to induce inflammatory responses and examined the effect of CBX2 on these responses in the absence of lipotoxicity. The results showed that CBX2 can directly affect the phosphorylation levels of NF‐κB/JNK (Figure S9C,D). Importantly, re‐expression of CBX2 in hepatocytes isolated from *Cbx2^LKO^* mice significantly attenuated lipid droplet formation and TG accumulation (Figure 8O,P).

Collectively, these findings demonstrate that CBX2 alleviates PA/OA‐induced lipid accumulation, inflammation, and insulin resistance in hepatocytes by simultaneously suppressing lipogenesis and enhancing fatty acid oxidation.

### 3.9 CBX2 Raises Intracellular Ca^2+^ to Drive CaMKK2‐AMPK Activation and Rebalance Lipid Metabolism

To further elucidate the molecular mechanism underlying CBX2‐mediated protection against hepatic lipid metabolic disorders, RNA‐seq was performed on primary hepatocyte overexpressing CBX2. Kyoto Encyclopedia of Genes and Genomes (KEGG) pathway analysis revealed that *Cbx2*‐regulated genes were significantly enriched in calcium and cAMP signaling pathways (Figure 9A). In line with this, CBX2 overexpression elevated cytosolic Ca^2+^ levels, whereas CBX2 knockdown decreased them (Figure 9B,C). Re‐expression of CBX2 in hepatocytes derived from CBX2‐knockout mice partially restored intracellular Ca^2+^ concentrations (Figure 9D).

**FIGURE 9:**
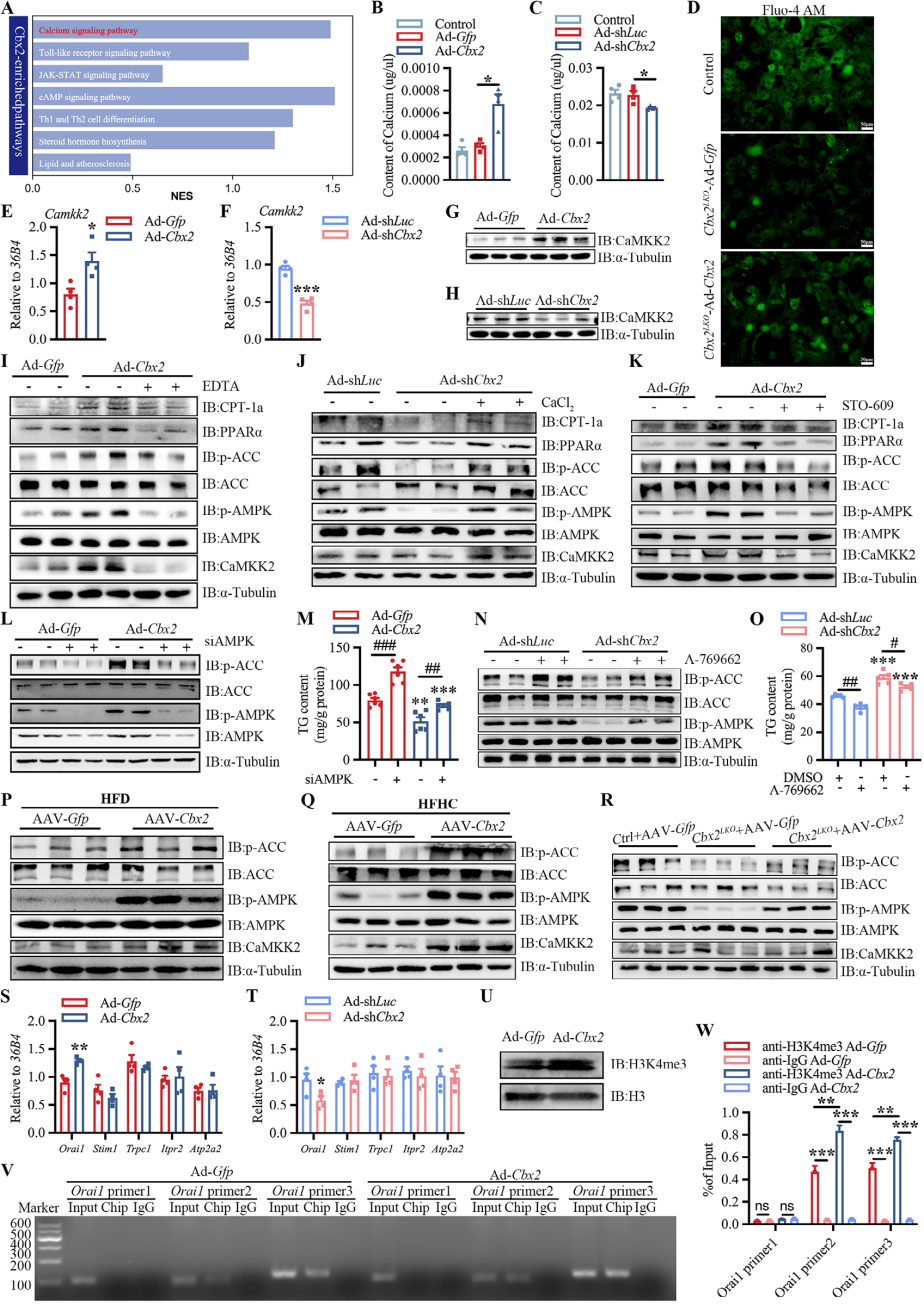
CBX2 regulates the AMPK signaling pathway via CaMKK2 by modulating intracellular Ca^2+^ levels. (A) KEGG pathway enrichment analysis of differentially expressed genes following CBX2 overexpression in primary mouse hepatocytes. (B, C) Quantification of intracellular Ca^2+^ content showing that CBX2 overexpression increased (B), whereas CBX2 knockdown decreased (C), intracellular Ca^2+^ levels in primary hepatocytes. (D) Representative images of Fluo‐4 AM staining demonstrating intracellular Ca^2+^ changes in primary mouse hepatocytes. (E) RT‐qPCR analysis showing that CBX2 overexpression upregulated *Camkk2* gene expression. (F) RT‐qPCR analysis showing that CBX2 knockdown reduced *Camkk2* gene expression. (G) Western blot analysis showing that CBX2 overexpression upregulated CaMKK2 protein expression. (H) Western blot analysis showing that CBX2 knockdown reduced CaMKK2 protein levels. (I–K) Western blot analyses of AMPK signaling‐related proteins in primary hepatocytes under different CBX2 expression conditions. (L) CBX2 overexpression enhanced phosphorylation of AMPK and its downstream target ACC; this effect was abolished by siAMPK. (M) Quantification of intracellular TG content in primary hepatocytes infected with Ad‐*Gfp* or Ad‐*Cbx2* for 24 h and subsequently treated with siAMPK. (N) CBX2 knockdown reduced phosphorylation of AMPK and ACC, which was reversed by the AMPK activator A‐769662 (10 µM). (O) Intracellular TG content in primary hepatocytes infected with Ad‐sh*Luc* or Ad‐sh*Cbx2* for 48 h and then treated with A‐769662 for 3 h. (P‐R) Western blot analyses of CaMKK2 expression and phosphorylation levels of AMPK and ACC in liver tissues from HFD‐induced obese mice (P), HFHC‐induced chronic inflammation mice (Q), and *Cbx2^LKO^* mice (R) after CBX2 overexpression. (S,T) RT‐qPCR analysis of the effects of CBX2 on Calcium channel‐related genes. (U) Western blot analysis of the effect of CBX2 overexpression on histone H3K4me3. (V) Representative ChIP‐qPCR electrophoresis image. (W) ChIP‐qPCR quantification results. Data are presented as mean ± SD. *Indicates statistical significance compared to Ad‐*Gfp* *p<0.05, **p<0.01, ***p<0.001. ^#^Indicates statistical significance compared to DMSO group. ^#^ *p* < 0.05, ^##^ *p* < 0.01, ^###^ *p* < 0.001.

Calcium signaling is known to regulate the calcium/calmodulin‐dependent protein kinase kinase 2 (CaMKK2), an upstream activator of AMPK, and a central energy sensor coordinating lipid metabolism [23, 24]. RT‐qPCR and western blot analysis showed that CBX2 overexpression increased CaMKK2 expression, whereas CBX2 knockdown reduced it (Figure 9E–H), without markedly altering phosphorylation level of CaMKK2 (Figure S10A). Furthermore, treatment with MG132 and CHX did not affect CaMKK2's expression (Figure S10B).

To verify whether CBX2 influences AMPK activation through calcium‐dependent regulation of CaMKK2, intracellular Ca^2+^ levels were manipulated using EDTA (Ca^2+^ chelator) or CaCl_2_ [25, 26]. In hepatocytes overexpressing CBX2, EDTA treatment abolished the CBX2‐induced upregulation of CaMKK2, phosphorylation of AMPK and ACC, and expression of fatty acid oxidation markers PPARα and CPT‐1a (Figure 9I). Conversely, CaCl_2_ supplementation restored CaMKK2 expression, AMPK/ACC phosphorylation, and PPARα/CPT‐1a expression suppressed by CBX2 knockdown (Figure 9J). Furthermore, pharmacological blockade of CaMKK2 using STO‐609 [27] abrogated CBX2‐induced AMPK and ACC phosphorylation and downstream gene expression (Figure 9K). Further validation with siAMPK demonstrated that genetic knockdown of AMPK abolished the promotive effects of CBX2 on AMPK and ACC phosphorylation, thereby restoring intracellular TG accumulation (Figure 9L,M). Consistent with the genetic results, the AMPK inhibitor compound C [28] exerted the same effect (Figure S10C,D). In contrast, activating AMPK with A‐769662 [29] rescued the impaired AMPK and ACC phosphorylation and lipid metabolic defects caused by CBX2 silencing (Figure 9N,O).

In vivo, hepatic overexpression of CBX2 significantly enhanced CaMKK2 expression and phosphorylation of AMPK and ACC in HFD‐ and HFHC‐fed mice relative to controls (Figure 9P,Q). Consistently, re‐expression of CBX2 in CBX2‐deficient livers restored CaMKK2 levels and AMPK/ACC phosphorylation (Figure 9R).

Nevertheless, the mechanism by which CBX2 elevates intracellular Ca^2+^ levels to trigger the above regulatory cascade remains unclear. To address how CBX2 modulates intracellular Ca^2+^ homeostasis and subsequently activates the downstream CaMKK2‐AMPK signaling axis, we screened core calcium channel genes in hepatocytes. RT‐qPCR analysis demonstrated that CBX2 overexpression significantly upregulated the transcription of *Orai1*, an essential constituent of the store‐operated calcium entry machinery. Conversely, CBX2 knockdown reduced *Orai1* expression (Figure 9S,T). These data indicate that *Orai1* may act as a critical downstream effector linking CBX2 to intracellular Ca^2+^ regulation. Given that CBX2 is a chromatin‐associated regulatory factor, we further explored whether CBX2‐mediated regulation of *Orai1* via epigenetic modulation. Western blot analysis showed that CBX2 overexpression markedly increased the level of H3K4me3, an active transcription‐associated histone mark (Figure 9U). To determine whether H3K4me3 enrichment occurred at the *Orai1* regulatory region, we performed ChIP‐qPCR targeting the *Orai1* promoter. Compared with *Ad‐Gfp* control group, CBX2 overexpression significantly enhanced H3K4me3 enrichment at specific regions of the *Orai1* promoter, especially the fragments amplified by primer 2 and primer 3 (Figure 9V,W).

Collectively, these findings indicate that CBX2 promotes *Orai1* transcriptional activation by facilitating H3K4me3 deposition at its promoter. We herein identify a previously characterized Ca^2+^/CaMKK2/AMPK signaling axis as a key downstream pathway through which CBX2 regulates hepatic lipid metabolism, promoting fatty acid oxidation while suppressing lipid accumulation and inflammation.

## 4 Discussion

MASLD is primarily driven by metabolic derangements that promote excessive hepatic lipid accumulation. A subset of patients progress to MASH, which can lead to hepatic fibrosis, cirrhosis, and hepatocellular carcinoma [30]. Although considerable progress has been made in elucidating the pathogenesis of MASLD and MASH, the molecular mechanisms that govern disease progression remain incompletely defined.

In this study, reanalysis of published RNA‐seq datasets revealed a marked reduction of *Cbx2* expression in the livers of MASLD mouse models. Consistent with these findings, RT‐qPCR and western blot analyses confirmed that hepatic CBX2 levels were significantly decreased in *db/db*, *ob/ob*, and HFD‐ or HFHC‐fed mice. Functionally, hepatic CBX2 overexpression alleviated HFD/HFHC‐induced hepatic lipid accumulation, IR and inflammation, whereas CBX2 knockdown or liver‐specific deletion exacerbated these phenotypes. Conversely, restoration of hepatic CBX2 expression normalized body weight gain, improved insulin sensitivity, and prevented MASLD development in *Cbx2^LKO^* mice. Mechanistically, CBX2 elevates intracellular Ca^2+^ concentrations in hepatocytes, thereby activating the CaMKK2‐AMPK signaling axis to suppress DNL and attenuate inflammation.

Further analyses demonstrated that CBX2 overexpression suppressed the expression of key DNL enzymes, including FAS, ACC, and SCD‐1, while concomitantly upregulating PPARα and CPT‐1a, two master regulators of mitochondrial β‐oxidation [21]. Consequently, hepatic CBX2 overexpression reduced fatty acid synthesis and enhanced fatty acid catabolism, whereas *Cbx2^LKO^* mice challenged with an HFD exhibited aggravated IR, steatosis, and inflammation‐all of which were completely reversed by hepatic CBX2 re‐expression.

Given that chronic inflammation underlies MASLD progression and promotes the shift from simple steatosis to MASH [31], we next examined the impact of CBX2 on inflammatory signaling. CBX2 overexpression markedly attenuated HFD‐ or HFHC‐induced elevations in serum ALT, AST, and TNFα levels, whereas *Cbx2^LKO^* significantly exacerbated these parameters. Consistently, F4/80 immunostaining revealed reduced macrophage infiltration and decreased expression of inflammation‐related genes in CBX2 overexpressing livers, whereas CBX2 deletion promoted macrophage recruitment. Moreover, CBX2 overexpression inhibited HFD‐induced phosphorylation of NF‐κB and JNK, while CBX2 deficiency further enhanced their activation. Similar anti‐inflammatory effects were observed in *db/db* and HFHC‐fed mice, reinforcing the notion that CBX2 mitigates hepatic inflammation in the context of metabolic stress.

Notably, we also observed that CBX2 overexpression significantly downregulated PLIN2, whereas CBX2 deletion enhanced its expression. PLIN2, a lipid‐droplet‐associated protein abundantly expressed in non‐adipose tissues such as the liver [32]. Previous studies have shown that PLIN2 deletion reduces HFD‐induced hepatic TG accumulation and obesity, and that liver‐specific PLIN2 ablation alleviates steatosis and inflammation [33]. Moreover, PLIN2 can be phosphorylated by AMPK, facilitating its degradation via chaperone‐mediated autophagy [34]. The precise mechanism by which CBX2 regulates PLIN2 expression warrants further investigation, but this finding suggests a potential link between CBX2, AMPK activation, and lipid droplet turnover.

During MASLD progression, hepatic fatty acid accumulation, IR, and chronic inflammation are interdependent pathological processes that mutually reinforce one another, establishing a self‐perpetuating cycle that drives disease advancement [35, 36, 37]. Excess fatty acids activate inflammatory pathways, inflammation exacerbates IR, and IR further promotes lipid accumulation. Therapeutic strategies that disrupt this cycle could therefore effectively halt disease progression. Our findings indicate that CBX2 overexpression simultaneously reduces lipid synthesis, improves insulin sensitivity, and dampens inflammatory responses, thereby breaking this vicious cycle and mitigating hepatic steatosis.

Transcriptomic profiling of primary hepatocytes infected with Ad‐*Cbx2* versus Ad‐*Gfp* revealed that CBX2‐regulated genes were significantly enriched in calcium signaling pathways. Consistent with this, CBX2 overexpression increased intracellular Ca^2+^ levels, whereas CBX2 knockdown reduced them. Calcium ions are essential second messengers in hepatocytes that regulate energy metabolism and lipid homeostasis [38]. CaMKK2, a key calcium effector protein, senses intracellular Ca^2+^ fluctuations and activates downstream signaling cascades [39]. We observed that CBX2 overexpression increased CaMKK2 expression, while CBX2 knockdown reduced it. The CaMKK2‐AMPK axis serves as a central metabolic hub linking Ca^2+^ signaling to the cellular energy state [39, 40], and its activation has been shown to ameliorate fatty liver disease [41, 42].

AMPK activation is mediated by three upstream kinases‐LKB1, CaMKK2, and TAK1‐each responding to distinct stimuli [43]. Once activated, AMPK phosphorylates and inhibits ACC, leading to reduced malonyl‐CoA levels, decreased lipid synthesis, and enhanced mitochondrial β‐oxidation [44]. Hepatic AMPKα overexpression in diabetic mice reduces lipogenic gene expression and hepatic TG accumulation [45]. Our data demonstrate that CBX2 overexpression elevates intracellular Ca^2+^, enhances CaMKK2 expression, and increases AMPKα phosphorylation, which collectively suppress lipogenesis while promoting fatty acid oxidation. Pharmacological inhibition of AMPK with Compound C abolished the CBX2‐induced reduction in TG content, whereas AMPK activation with A‐769662 rescued the lipid accumulation phenotype of CBX2‐deficient hepatocytes. In vivo, hepatic CBX2 restoration in HFD‐, HFHC‐, and CBX2‐knockout mice consistently elevated CaMKK2 levels and AMPK phosphorylation, corroborating the functional link between CBX2 and Ca^2+^‐CaMKK2‐AMPK signaling. The molecular mechanism by which CBX2 modulates intracellular Ca^2+^ homeostasis remains poorly defined. To address this issue, we profiled the expression of calcium channel genes and identified *Orai1* as a potential downstream mediator of CBX2‐induced Ca^2+^ elevation. ORAI1 is a highly conserved plasma membrane calcium channel protein and serves as the essential pore‐forming component of the store‐operated calcium entry (SOCE) pathway [46]. Upon depletion of endoplasmic reticulum (ER) Ca^2+^ stores, the ER calcium sensor STIM1 undergoes conformational changes and translocates toward the ER‐plasma membrane junctions, where it interacts with ORAI1 to activate Ca^2+^ influx from the extracellular space [47]. Notably, this STIM1‐ORAI1‐mediated Ca^2+^ entry represents one of the major mechanisms controlling intracellular calcium signaling and regulates diverse cellular processes, including hepatic metabolism, inflammation responses, and cell survival.

Beyond metabolic regulation, accumulating evidence suggests that CBX2 also modulates gene expression through epigenetic mechanisms [48, 49]. CBX2 recognizes H3K27me modifications and recruits PRC complexes to target loci, promoting chromatin compaction and transcriptional repression [14]. However, noncanonical roles of CBX2 have also been reported. For example, CBX2 can suppress interferon signaling independently of PRC association [50] and can recruit the demethylase Jmjd3 to the *Ifnb* promoter to remove repressive H3K27me3 marks, thereby enhancing interferon‐β transcription [51]. Similarly, CBX4 suppresses Runx2 expression and inhibits colorectal cancer metastasis through an HDAC3‐dependent mechanism [52]. Our results demonstrated that CBX2 overexpression elevated H3K4me3 levels, which is a canonical promoter‐associated histone modification that marks transcriptionally active chromatin regions and facilitates gene expression by promoting a permissive chromatin state [53]. ChIP‐qPCR results further confirmed that CBX2 overexpression enhanced H3K4me3 enrichment at specific regions of the *Orai1* promoter, accompanied by increased *Orai1* transcription. Collectively, these findings suggest that CBX2 drives *Orai1* transcriptional activation through modulation of active histone modifications, providing a potential molecular explanation for how CBX2 regulates intracellular Ca^2+^ signaling. Our findings identify a previously unrecognized CBX2‐*Orai1*‐Ca^2+^‐CaMKK2‐AMPK signaling axis linking chromatin‐associated regulation with hepatic metabolic homeostasis.

Nonetheless, our study has limitations. Further studies are required to determine the precise molecular partners involved in CBX2‐mediated chromatin remodeling at the *Orai1* locus, and whether its canonical PRC‐mediated functions contribute to hepatic lipid regulation requires further investigation.

## 5 Conclusions

In conclusion, our findings identify CBX2 as a critical regulator of hepatic lipid metabolism that promotes H3K4me3 enrichment at the *Orai1* promoter to facilitate *Orai1* transcriptional activation, thereby increasing intracellular Ca^2+^ levels and activating the Ca^2+^‐CaMKK2‐AMPK signaling axis to inhibit lipogenesis and inflammation while enhancing fatty acid oxidation. These results provide new mechanistic insights into the metabolic regulation of MASLD and suggest that targeting CBX2 or its downstream signaling pathway may represent a promising therapeutic strategy.

## Author Contributions

**Xiang Wei**, **Li Lin**, **Xiuyun Wang** and **Hui Du** contributed equally to this work. **Huabing Zhang**, **Yongsheng Chang**, **Wei Chen** and **Yinliang Qi** conceived and designed the experiments. Xiang Wei, Li Lin, Xiuyun Wang, Hui Du, **Menglong Zhang**, **Kechao Tang**, and Jinya Gao performed the experiments. **Xu Wang** completed the data analysis RNA sequencing. **Han Gao**, **Xinrui Xie**, **Zhenglin Wang** and **Siwei Huang** collected data. Huabing Zhang, **Lei Zhang**, Xiuyun Wang and **Fang Gu** provided funding. Xiang Wei, Huabing Zhang and **Gong‐Hong Wei** drafted the original manuscript. All authors contributed to and have approved the final manuscript.

## Funding

This study was supported by the National Natural Science Foundation of China (Nos. 82370878, 82200975, and 82300659), the Anhui Province Excellent Research and Innovation Team Project (No. 2025AHGXZK10011), the Anhui Science Fund for Distinguished Young Scholars (No. 2208085J45), the Natural Science Foundation of Anhui Province (No. 2308085QH247) and Natural Science Foundation of Colleges and Universities in Anhui Province (No. 2022AH050765).

## Conflicts of Interest

The authors declare no conflicts of interest.

## Supporting information




**Supporting File**: advs78372‐sup‐0001‐SuppMat.docx.

## Data Availability

The data that support the findings of this study are available on request from the corresponding author. The data are not publicly available due to privacy or ethical restrictions.
