# Supplementary material for: Hepatocyte CBX2 Safeguards Hepatic Lipid Homeostasis by Elevating Ca2+‐CaMKK2‐AMPK Signaling to Counter MASLD
Source: Adv Sci (Weinh). 2026 Oct 18:e78372. Online ahead of print. doi: 10.1002/advs.78372 (PMC13677047; doi:10.1002/advs.78372)
Supplement: Supplementary file 1 [file ADVS-9999-e78372-s001.docx]

Supporting Information

Hepatocyte CBX2 safeguards hepatic lipid homeostasis by elevating Ca²⁺-CaMKK2-AMPK signaling to counter MASLD

Xiang Wei^1,2#^, Li Lin^1#^, Xiuyun Wang^1#^, Hui Du^8#^, Menglong Zhang^1^, Kechao Tang^1^, Han Gao^1^, Xinrui Xie^1^, Jingya Gao^1^, Xu Wang^6^, Zhenglin Wang^5^, Siwei Huang^5^, Lei Zhang^7^, Fang Gu^1^, Gong-Hong Wei^8,9^, Yinliang Qi^2^*, Wei Chen^5^*, Yongsheng Chang^4^*, Huabing Zhang^1,3^*

| Supplementary Table S1. Primer | | |
| --- | --- | --- |
| Mouse  (gene) | Forward Primer | Reverse Primer |
| *Cbx2* | 5’CCAGAAGAGAACATTTTGGACCCG’3 | 5’GCATCTGGTTCCTTGAGCTTG’3 |
| *Srebp-1c* | 5’GGAGCCATGGATTGCACATT’3 | 5’GGCCAGGGAAGTCACTGT’3 |
| *Fas* | 5’GTAAGTTCTGTGGCTCCAGAG’3 | 5’GCCCTCCCGTACACTCACTC’3 |
| *Acc* | 5’AGGAAGATGGCGTCCGCTCTG’3 | 5’GGTGAGATGTGCTGGGTCAT’3 |
| *Scd-1* | 5’CTGCACCTCCCTCCGGAAAT’3 | 5’TCCTCCAGACGTACTCCAGC’3 |
| *Pparα* | 5’ACAAGGCCTCAGGGTACCA’3 | 5’GCCGAAAGAAGCCCTTACAG’3 |
| *Cpt-1a* | 5’GAACCCCAACATCCCCAAAC’3 | 5’TCCTGGCATTCTCCTGGAAT’3 |
| *Cd36* | 5’TCCCTTGGCAACCAACCAC’3 | 5’TCCACCAGTTGCTCCACAC’3 |
| *Mttp* | 5’AGTGCAGTTCTCACAGTACCCGTT’3 | 5’AGCATATCGTTCTGGTGGAAGGGA’3 |
| *Plin2* | 5’CAATTTGTGGCTCCAGCTTC’3 | 5’CCCGTATTTGAGATCCGTGT’3 |
| *Ccl2* | 5’TCTGGACCCATTCCTTCTTG’3 | 5’AGGTCCCTGTCATGCTTCTG’3 |
| *Tnfα* | 5’CGTCAGCCGATTTGCTATCT’3 | 5’CGGACTCCGCAAAGTCTAAG’3 |
| *Il-1β* | 5’TGAAGTTGACGGACCCCAAAA’3 | 5’TGATGTGCTGCTGCGAGATT’3 |
| *Il-6* | 5’AGTTGCCTTCTTGGGACTGA’3 | 5’TCCACGATTTCCCAGAGAAC’3 |
| *Orai1* | 5’GGCTTCGCCATGGTAGCG’3 | 5’GCTCGTGGGGTGACTCTTTG’3 |
| *Stim1* | 5’CTCTCTCTGCTCTGCATCCG’3 | 5’GGGTCAAATCCCTCTGAGATCC’3 |
| *Trpc1* | 5’TGGCCAGTCCAGCTCTGATA’3 | 5’AACATTTTGCACTGACGGGC’3 |
| *Itpr2* | 5’CGAGGGTGATAATGTGAATGCTG’3 | 5’CTAGTGGCCGAAGTTGCTGA’3 |
| *Atp2a2* | 5’GAGACGCTCAAGTTTGTGGC’3 | 5’CAGACCACCAGGGGCATAAT’3 |
| siAMPK | 5’AUGAUGUCAGAUGGUGAAUUU’3 | 5’AAAUUCACCAUCUGACAUCAU’3 |
| *Orai1* primer1 | 5’CCATACGTGGGGCTATGTAGG’3 | 5’TCCTACATGGGCTTTGTGCT’3 |
| *Orai1* primer2 | 5’AAGTTCCTTAGCCCAGTGCC’3 | 5’AGATAGGCGCCAGGACCTAA’3 |
| *Orai1* primer3 | 5’TGCCACCTTGTTCCAAGGAG’3 | 5’GTCAGGCCTCGCTTCTCTTT’3 |
| *36B4* | 5’ATCCCTGACGCACCGCCGTGA’3 | 5’TGCATCTGCTTGGAGCCCACGTT’3 |
| flox | 5’TTTGTTTACGAGCCCACGGG’3 | 5’TACAAAGGAAGCCCTTTCCGG’3 |
| cre | 5’CCCAAAGTCGCTCTGAGTTGTTA’3 | 5’GGCTGGACCAATGTGAACATTG’3 |
| WT | 5’CCCAAAGTCGCTCTGAGTTGTTA’3 | 5’TCGGGTGAGCATGTCTTTAATCT’3 |

| Supplementary Table S2. Primary antibodies | | | |  |
| --- | --- | --- | --- | --- |
| Anti-body | Dilution ratio | Brands | Item No. | Country |
| CBX2 | 1:1000 | Proteintech | 15579-1-AP | Wuhan, China |
| AKT | 1:1000 | CST | #9272S | Boston, USA |
| p-AKT | 1:1000 | CST | #9271S | Boston, USA |
| GSK-3β | 1:1000 | CST | #9315S | Boston, USA |
| p-GSK-3β | 1:1000 | CST | #5558S | Boston, USA |
| AMPK | 1:1000 | CST | #5832 | Boston, USA |
| p-AMPK | 1:1000 | CST | #2535 | Boston, USA |
| ACC | 1:10000 | Proteintech | 67373-1-Ig | Wuhan, China |
| p-ACC | 1:1000 | CST | #11818 | Boston, USA |
| NF-κB | 1:1000 | CST | #8242T | Boston, USA |
| p-NF-κB | 1:1000 | CST | #3033T | Boston, USA |
| JNK | 1:1000 | CST | #9252 | Boston, USA |
| p-JNK | 1:1000 | CST | #4668 | Boston, USA |
| PEPCK | 1:5000 | Proteintech | 16754-1-AP | Wuhan, China |
| G6pase | 1:1000 | Abclonal | A21168 | Wuhan, China |
| PGC-1α | 1:1000 | Proteintech | 66369-1-lg | Wuhan, China |
| FAS | 1:1000 | Santa cruz | sc-74540 | Dallas, USA |
| SCD-1 | 1:1000 | Abclonal | A16429 | Wuhan, China |
| PLIN2 | 1:5000 | Proteintech | 15294-1-AP | Wuhan, China |
| F4/80 | 1:2000 | Proteintech | 28463-1-AP | Wuhan, China |
| CPT-1a | 1:1000 | Abcam | ab128568 | Cambridge, UK |
| PPARα | 1:1000 | Boster | BA1691 | Wuhan, China |
| CaMKK2 | 1:500 | Affinity | #DF4793 | Jiangsu, China |
| p-CaMKK2 | 1:500 | Affinity | #AF4487 | Jiangsu, China |
| FLAG | 1:5000 | Sigma | F3165 | Darmstadt, Germany |
| α-Tubulin | 1:1000 | CST | #2125 | Boston, USA |
| H3K4me3 | 1:1000 | Proteintech | 39060 | Wuhan, China |
| H3 | 1:1000 | Proteintech | 17168-1-AP | Wuhan, China |
| Goat Anti-Rabbit IgG | 1:10000 | Jackson | 111-035-003 | PA, USA |
| Goat Anti-Rabbit IgG | 1:5000 | Biomike | MK101A | Shanghai, China |

| Table 3. Reagent Information |  |  |  |
| --- | --- | --- | --- |
|  | Product Number | Brand | Country |
| PA | P9767 | Sigma Aldrich | Missouri, USA |
| OA | O7501 | Sigma Aldrich | Missouri, USA |
| glucose | 50-99-7 | Sangon Biotech | Shanghai, China |
| insulin | I8048 | Slarbio | Beijing, China |
| LPS | L6023 | Sigma Aldrich | Missouri, USA |
| DAB | ZLI-9017 | ZSGB-BIO | Beijing, China |
| Oil Red O | O8010 | Solarbio | Beijing, China |
| TG assay kit | E1013 | Applygen | Beijing, China |
| TC assay kit | E1015 | Applygen | Beijing, China |
| colorimetric calcium assay kit | S1063S | Beyotime | Shanghai, China |
| Fluo-4 AM | S1060 | Beyotime | Shanghai, China |
| TNFα ELISA kit | E-MSEL-M0002 | Elabscience | Wuhan, China |
| TRIzol | AC0101-A | Sparkjade | Shandong, China |
| First-Strand cDNA Synthesis Kit | RK20429 | Abclonal | Wuhan, China |
| SYBR Green | RK21203 | Abclonal | Wuhan, China |
| ECL kit | #17046 | ZENBIO | Chengdu, China |
| Histone Extraction Kit | PK10022 | Proteintech | Wuhan, China |

**Supplementary Figures**

**Supplementary Figure 1. The protein expression of hepatic gluconeogenesis-related genes (PGC-1α, PEPCK, and G6Pase) in HFD-induced obese mice treated with AAV-*Cbx2*.**

**Supplementary Figure 2. Hepatic CBX2 overexpression showed no significant effect on glucose and lipid metabolism in mice fed a normal chow diet.**

(A) RT-qPCR analysed the expression of *Cbx2* in liver tissue from normal diet (ND) mice; (n=8). (B) There was no significant change in body weight in ND mice following CBX2 overexpression. (C, D) GTT (C) and ITT (D) analyzed glucose tolerance and insulin resistance in ND mice after CBX2 overexpression. (E) Hepatic lipid accumulation in ND mice after CBX2 overexpression*.* (F, G) Hepatic TG F) and TC (G) levels in ND mice. (H-M) Serum TG (H), TC (I), FFA (J), ALT (K), AST (L), and TNFα (M) levels. Data are presented as mean ± SD. *Indicates statistical significance compared to AAV-*Gfp*, **p*<0.05，***p*<0.01, ****p*<0.001.

**Supplementary Figure 3. Hepatic CBX2 overexpression improves glucose tolerance and insulin sensitivity in *db/db* mice.**

(A) RT-qPCR was used to detect the expression of *Cbx2* in liver tissues; (*n*=6). (B, C) Gross morphology (B) and body weight (C) of representative mice in the two groups. (D) Blood glucose levels of *db/db* mice in the AAV-*Gfp* group and the AAV-*Cbx2* group. (E, F) GTT (E) and ITT (F) to detected glucose tolerance and insulin sensitivity in *db/db* mice following CBX2 overexpression. (G) Western blot assayed for insulin signaling pathway-related protein expression. (H) AKT and GSK-3β phosphorylation densitometry in (G). Data are presented as mean ± SD. *Indicates statistical significance compared to AAV-*Gfp*, **p*<0.05, ***p*<0.01, ****p*<0.001.

**Supplementary Figure 4. CBX2 overexpression attenuates hepatic steatosis and inflammation in *db/db* mice.**

(A) Representative liver morphology of the two groups. (B) Liver H&E staining, Oil Red O staining and F4/80 staining were used to observe liver lipid accumulation and macrophage infiltration (×400, scale bar: 20 µm). (C) Liver weight to body weight ratio in each mouse group. (D, E) The contents of TG (D) and TC (E) in the liver. (F-H) The contents of TG (F), TC (G) and FFA (H) in serum. (I, J) The contents of ALT (I) and AST (J) in serum. (K) RT-qPCR detected the expression of inflammation-related genes. (L) Western blot analyed the phosphorylation levels of JNK and NF-κB. (M, N) RT-qPCR (M) and western blot (N) detected the expression of fatty acid metabolism-related genes in the livers of *db/db* mice. Data are presented as mean ± SD. *Indicates statistical significance compared to AAV-*Gfp*, **p*<0.05, ***p*<0.01, ****p*<0.001.

**Supplementary Figure 5. The overexpression of CBX2 in the liver of mice with chronic inflammation induced by HFHC diet.**

(A, B) RT-qPCR (A) and western blot (B) detected the expression of CBX2 in the livers of HFHC-induced chronic inflammation mice; (*n*=8). Data are presented as mean ± SD. *Indicates statistical significance compared to AAV-*Gfp*, **p*<0.05, ***p*<0.01, ****p*<0.001.

**Supplementary Figure 6. Liver-specific CBX2 knockdown exacerbates glucose tolerance and insulin resistance in HFD-induced obese mice.**

(A, B) RT-qPCR (A) and western blot (B) showed a significant reduction in CBX2 in the livers of HFD-induced obese mice; (*n*=8). (C, D) CBX2 knockdown increased body weight (C) and blood glucose (D) in HFD-induced obese mice. (E, F) CBX2 knockdown impaired glucose tolerance (E) and insulin sensitivity (F) in HFD-induced obese mice. (G) Western blot detected the phosphorylation of AKT and GSK-3β. (H) AKT and GSK-3β phosphorylation densitometry in (G). Data are presented as mean ± SD. *Indicates statistical significance compared to AAV-Ctrl, **p*<0.05，***p*<0.01, ****p*<0.001.

**Supplementary Figure 7. CBX2 knockdown aggravated HFD-induced hepatic steatosis and inflammation.**

(A, B) Representative liver morphology (A) and liver weight (B) of each group. (C) Liver sections were stained with H&E and Oil Red O staining to observed the liver injury and lipid accumulation in the liver (×400, scale bars: 20 µm). (D, E) Hepatic TG (D) and TC (E) contents. (F-H) Serum TG (F), TC (G), and FFA (H) levels. (I-K) Serum ALT (I), AST (J), TNFa (K) levels. (L) Histological F4/80 staining of liver paraffin sections (×400, scale bars, 20 μm) in the AAV-Ctrl group and AAV-sh*Cbx2* group mice. (M, N) RT-qPCR (M) and western blot (N) detected the expression of fatty acid metabolism-related genes in the livers of HFD-induced obesity. (O) RT-qPCR detected the expression of inflammation-related genes. (P) Western blot analysis of JNK and NF-κB protein and phosphorylation levels in the hepatic of AAV-Ctrl and AAV-sh*Cbx2* groups. (Q) NF-κB and JNK phosphorylation densitometry in (P). Data are presented as mean ± SD. *Indicates statistical significance compared to AAV-Ctrl, **p*<0.05, ***p*<0.01, ****p*<0.001.

**Supplementary Figure 8. Generation of liver-specific CBX2 knockout mice**.

(A) Genotyping of *Cbx2^LKO^* mice. (B) Genotyping of *Cbx2^fiox/flox^* mice. (C) Expression of *Cbx2* in various tissues of liver-specific *Cbx2* knockout mice. (D, E) RT-qPCR (D) and western blot (E) analysis of CBX2 expression. Data are presented as mean ± SD. *Indicates statistical significance compared to Ctrl or Ctrl+AAV-*Gfp*, ^#^Indicates statistical significance compared to *Cbx2^LKO^*+AAV-*Gfp*, * ^or #^*p*<0.05, ** ^or ##^*p*<0.01, *** ^or ###^*p*<0.001.

**Supplementary Figure 9. The effect of CBX2 on the inflammatory response under conditions free of lipid toxicity.**

(A) After treatment with adenovirus Ad-*Cbx2* for 24 hours, the levels of CBX2 significantly increased in primary mouse hepatocytes. (B) Successful knockdown of CBX2 expression in mouse primary hepatocytes by using adenovirus Ad-sh*Cbx2*. (C) CBX2 overexpression reduced LPS-induced phosphorylation of NF-κB and JNK in primary hepatocytes. (D) CBX2 knockdown increased LPS-induced phosphorylation of NF-κB and JNK in primary hepatocytes.

**Supplementary Figure 10. Overexpression of CBX2 affects lipid synthesis via the AMPK signaling pathway.**

(A) Western blot detected the phosphorylation of CaMKK2. (B) Analysis of CaMKK2 changes following treatment with MG132 (25 μM) and CHX (50 mg/ml). (C) CBX2 overexpression enhanced phosphorylation of AMPK and its downstream target ACC; this effect was abolished by the AMPK inhibitor Compound C (10μM). (D) Quantification of intracellular TG content in primary hepatocytes infected with Ad-Gfp or Ad-*Cbx2* for 24 h and subsequently treated with Compound C for 12 h. Data are presented as mean ± SD. *Indicates statistical significance compared to Ad-*Gfp* **p*<0.05, ***p*<0.01, ****p*<0.001. ^#^Indicates statistical significance compared to DMSO group. ^#^*p* < 0.05, ^##^*p* < 0.01, ^###^*p* < 0.001.
